# Techno-economic optimization of a hybrid microgrid with integrated backup storage and vehicle-to-grid functionality

**DOI:** 10.1038/s41598-026-64181-9

**Published:** 2026-08-05

**Authors:** Noha Nabil Abd-Elhady, Mohammed Fathy Ahmed, Salama Abu-Zaid, Taghreed Said

**Affiliations:** 1 Electrical Engineering Department, Faculty of Engineering, Al-Azhar University, Cairo, Egypt; 2 Electrical Engineering Department, Higher Technological Institute, 10th of Ramadan City, Egypt

**Keywords:** Cloud drift optimization (CDO) algorithm, Hydrogen energy storage system, V2G-equipped microgrid, Optimization of combined energy storage technologies, Energy stability in isolated regions, Energy science and technology, Engineering

## Abstract

This study presents the techno-economic optimization of a hybrid backup system integrated within an off-grid microgrid framework with electric vehicle (EV) grid-interaction capability. A real-world case study from a remote region in Egypt is used to evaluate system performance under realistic operating conditions. The optimization problem is formulated to minimize the net present cost (NPC) while ensuring system reliability using a penalty-based loss of power supply probability (LPSP). The system integrates photovoltaic (PV), wind turbines (WT), battery energy storage systems (BESS), hydrogen energy storage systems (HESS), and EVs. The novelty of this work lies in the development of a coordinated multi-storage energy management strategy that integrates BESS, HESS, and constrained EV participation within a unified optimization framework. The results show that the BESS-only configuration achieves the lowest cost (NPC ≈ $20.33 billion), while the PV/WT/BESS/HESS configuration results in the highest cost (NPC ≈ $25.48 billion). The proposed PV/WT/BESS/HESS/EV configuration provides a balanced solution with an NPC of approximately $22.79 billion while maintaining near-zero LPSP. EV integration enhances system flexibility, reducing the required BESS capacity by 44.8% relative to the HESS-only configuration, while also lowering reliance on hydrogen-based long-term storage, thereby mitigating the high capital costs associated with extended-duration storage components. These findings demonstrate that coordinated multi-storage management significantly improves the techno-economic viability and operational resilience of large-scale off-grid microgrids in remote regions.

## Introduction

### General overview

Energy systems rely heavily on fossil fuels, which are finite resources associated with harmful carbon emissions that contribute significantly to global warming. Their depletion and increasing scarcity have led to rising energy prices and growing economic concerns^1,2^.

The continued dependence on fossil fuels can hinder sustainable development and contribute to economic and environmental instability, prompting a global shift toward cleaner alternatives such as renewable energy sources and electric vehicles.

Energy is a key driver of economic growth; however, conventional generation technologies are major contributors to environmental degradation and climate change ^3–5^. Consequently, significant efforts have been directed toward the adoption of renewable energy sources (RESs), which are characterized by sustainability, widespread availability, and minimal environmental impact^6–8^.

Among these, photovoltaic (PV) systems and wind turbines (WT) are considered highly promising due to their economic viability and near-zero carbon emissions^9,10^. However, their inherent intermittency and stochastic behavior pose challenges to system stability and reliability^11^. To overcome these limitations, hybrid renewable energy systems (HRESs) incorporating energy storage technologies have been widely proposed, as they can mitigate fluctuations and enhance supply reliability^12,13^. In this context, renewable-based microgrids have emerged as a viable solution for reducing emissions and dependence on fossil fuels.

Egypt has substantial solar and wind energy potential; nevertheless, remote and desert regions continue to face inadequate electricity access due to limitations in grid expansion ^14,15^. The Integrated Sustainable Energy Strategy 2035 aims to increase the share of renewable energy, where renewable-based microgrids supported by advanced energy storage technologies are expected to play a pivotal role^16^. Accordingly, such systems represent a strategic pathway toward improving long-term energy security, environmental sustainability, and economic development.

### Renewable energy potential in Egypt

Egypt possesses abundant and diverse natural energy resources, including significant reserves of oil and natural gas, alongside substantial renewable energy potential. It is characterized by the availability of various energy resources, including coal, oil, natural gas, and other fossil fuels. The natural gas reserves in Egypt are estimated to be approximately 77,200 billion cubic meters. Oil reserves totaled around 4189 billion gallons^17^. In Egypt, natural gas and oil are considered the most dependable sources for power generation. They account for almost 90% of all power generated. Natural gas accounts for around 77.37% of energy generation, while other fossil fuels account for 12.64%. Egypt generates the remaining electricity using renewable energy sources^16^.

Egypt has one of the largest economies in Africa and plays a significant role in global energy transmission. Despite this, Estimates suggest that Egypt’s continued reliance on fossil-based energy sources could lead to a 125% increase in carbon dioxide emissions between 2012 and 2035^18^. Egypt supported the Kyoto Protocol, recognizing the need for global collaboration. Therefore, it should prioritize renewable energy technology to meet its energy needs. Egypt’s renewable energy sector has untapped potential for sustainable electricity generation.

Solar energy contributes approximately 1.9% to Egypt’s total electricity generation, positioning it as the second-largest source of renewable energy^18^. According to ref^19^., In Africa, Egypt is the second-largest solar power producer after South Africa. Egypt ranks thirty-first in worldwide solar energy generation. Globally, many countries are increasingly meeting their electricity demand through wind energy conversion systems (WECSs), reflecting the growing role of wind power in sustainable energy transitions. Globally, the United States ranks first in wind energy production, whereas Asia generates more wind power than Europe. China leads the Asian wind energy sector, accounting for over 70%^18^. Egypt’s national electrical infrastructure has recently grown significantly and rapidly.

Many power plants have been built to improve grid dependability and permit developments into rural locations. Egypt is aggressively creating renewable energy projects, some of which are already finished. Renewable energy projects in Egypt are distributed across several regions and technologies with different installed capacities. Hydropower plants represent a significant portion of renewable generation. The High Dam in Aswan has a capacity of about 2100 MW, followed by other stations in Aswan (280 MW and 270 MW). Additional hydropower facilities are located in Isna, Qena (86 MW), Naga Hamady, Qena (64 MW), and Asyut (32 MW).

Wind energy projects are mainly concentrated along the Red Sea coast. Large wind farms operate in the Gulf of El Zayt, including 240 MW (Project 1), 220 MW (Project 2), and 120 MW (Project 3) located in Ras Gharib city. Other wind energy conversion system (WECS) installations include 545 MW at Zaafarana in Ras Gharib, as well as 250 MW in the Gulf of Suez and 250 MW in Ras Gharib.

Concentrated solar power (CSP) and solar photovoltaic projects also contribute to Egypt’s renewable portfolio. The Benban Solar Park in Aswan, with a capacity of 1465 MW, is one of the largest solar installations. Additional projects include 250 MW and 26 MW plants in Kom Umbu (Aswan), 50 MW in Zaafarana (Ras Gharib), 20 MW in Al-Kuraymat (Giza), and 20 MW in Hurghada (Red Sea).

Several smaller photovoltaic systems are distributed in remote areas, such as Siwa (10 MW) in Matrouh, Marsa Alam (6 MW), Shalateen (5 MW), Halayeb (2 MW), and Abu-Ramad (2 MW) in the Red Sea region. Additional solar installations are located in Al-Farafra (5 MW), Darb Al-Arbaeen (0.5 MW), and Abu-Minqar (0.5 MW) in the New Valley, along with a 0.66 MW system at Cairo University in Giza.

In addition to these resources, bioenergy generation is utilized at Al-Gabal Al-Asfar in Qalyubia, with a capacity of approximately 10 MW^18^.

Egypt’s population distribution is highly uneven and is heavily concentrated in the Nile Valley and Delta regions, posing significant socioeconomic and infrastructural challenges. The government has long struggled to address population concentrations in the valley of the Nile and Delta areas. The government’s attempts to achieve balanced distribution of people are vital for long-term growth. Electrifying distant locations and encouraging development has several benefits. The government has implemented a comprehensive approach that involves both electrification and improvements in transportation infrastructure.

Electrification of rural regions is often associated with high costs and economic limitations. Isolated renewable energy–based microgrids provide a practical and sustainable alternative to main grid expansion, significantly enhancing both the availability and reliability of electricity supply. Establishing a reliable backup system is crucial for microgrids due to the intermittent nature of their power sources. Diesel generators are widely used; however, their environmental effect is a major problem. This study proposes and evaluates several environmentally friendly hybrid backup configurations, and their performance is comprehensively analyzed from techno-economic and reliability perspectives.

### Literature review

Integrating renewable energy sources with battery and hydrogen storage systems has been widely acknowledged as an effective approach to enhance system performance. For instance, studies^20,21^ focused on improving economic performance and cost optimization, while^22,23^ emphasized system reliability and operational flexibility in hybrid energy systems. Multi-objective optimization methods have also been extensively employed, where^24^ addressed cost–environment trade-offs.

In particular, PV–WT–BESS configurations have demonstrated strong techno-economic viability in remote areas, where studies^25,26^ focused on optimal sizing and cost-effective operation of hybrid renewable systems. In contrast, PV–wind–diesel–battery systems have been investigated in^27–29^, where^27^ focused on reducing fuel consumption through optimal dispatch strategies, while^28,29^ emphasized emission reduction and reliability improvement in hybrid configurations. Furthermore, recent studies conducted in Egypt^30,31^ have validated the feasibility of renewable-based hybrid systems for green hydrogen production and rural electrification, where^30^ addressed techno-economic feasibility, and^31^ highlighted environmental and sustainability benefits.

Recent advancements have also focused on sophisticated energy management techniques. Metaheuristic optimization approaches^32–34^ have been widely applied for system sizing and optimization, where^32,33^ focused on improving convergence characteristics, while^34^ emphasized solution accuracy and robustness. Meanwhile, artificial intelligence-based methods such as deep reinforcement learning (DRL) and model predictive control (MPC)^35,36^ have been utilized to enhance system coordination, where^35^ addressed adaptive control strategies, and^36^ focused on real-time operational efficiency and system flexibility.

In addition, vehicle-to-grid (V2G) integration has been shown to enhance system reliability and reduce dependence on stationary storage^37^, while hydrogen-based storage systems contribute to long-term energy autonomy and reduced life-cycle costs^38^.

Recent studies have also explored techno-economic optimization and system reliability challenges in renewable-integrated power systems, particularly in weak-grid conditions. Advanced hybrid optimization approaches and control strategies have been proposed to enhance system stability, reliability, and economic performance under high renewable penetration. These studies highlight the growing importance of integrating reliability considerations within techno-economic frameworks, especially in systems with increasing electrification and EV participation^39–41^.

Recent studies have also focused on advanced optimization techniques for the optimal sizing and deployment of battery energy storage systems (BESS), demonstrating improved system performance, cost efficiency, and reliability. In particular, recent optimization-based approaches have shown significant potential in enhancing storage integration within renewable energy systems^42,43^.

Recent studies have also explored advanced optimization frameworks for large-scale and integrated energy systems. For instance, multi-figure-of-merit optimization approaches have been proposed to simultaneously address economic, technical, and environmental objectives in sustainable power systems. In addition, optimal operation strategies for electric vehicle charging stations in open electricity markets have been investigated, highlighting the growing role of EVs in enhancing system flexibility and economic performance^44,45^.

Recent studies have extensively investigated optimization and control strategies for improving the performance of power systems and renewable-integrated networks. A considerable body of work has focused on optimal placement and sizing of distributed generation (DG) units, reactive power compensation, and network reconfiguration using advanced metaheuristic and hybrid optimization techniques. Other studies have explored coordinated operation strategies in distribution systems and microgrids, as well as intelligent and machine learning-based approaches for enhancing system efficiency and flexibility under varying operating conditions. These efforts have contributed significantly to improving techno-economic performance and operational stability in modern energy systems^46–50^. Previous research has addressed various optimization challenges in distribution networks and microgrids. To overcome the premature convergence of BPSO, a hybrid Chaotic BPSO (CBPSO) was developed for DNR under variable loads, achieving up to 35% loss reduction^51^.For DSR, an enhanced WLSSA was introduced, outperforming conventional algorithms by achieving a 75.75% loss reduction on a real Iraqi 33-bus system^52^. For reactive power compensation, a WSSA was proposed for optimal capacitor placement, simultaneously minimizing power loss (34.81%) and annual costs (29.08%), yielding over $30,965 in annual savings^53^.In the context of smart grids, a DRL-based EMS using DDPG was developed for multi-HMG coordination, reducing grid imports by 21% while enhancing system resilience^54^.

The increasing penetration of electric vehicle (EV) charging stations has introduced additional challenges to power system operation, including increased load demand, voltage fluctuations, and higher energy losses. Recent studies have investigated these impacts and proposed integrated solutions to enhance system performance. For instance, the work in^55^ analyzed the role of renewable energy sources and reactive power compensation in reducing energy losses in distribution systems with EV charging stations. Similarly^56^, presented an optimal economic framework for integrating EV charging infrastructure with renewable energy technologies and capacitor banks, demonstrating improved economic performance and voltage stability.

In addition, recent research has focused on the optimal coordination of energy storage systems with renewable generation to minimize total system cost and improve reliability. The study in^57^ highlighted the effectiveness of combining battery storage and renewable energy in reducing operational costs and enhancing system efficiency.

These findings emphasize the importance of coordinated energy m\anagement strategies that consider the interaction between EV charging demand, renewable energy generation, and storage systems, which directly motivates the proposed framework in this study.

These studies emphasize the importance of integrating advanced optimization techniques and EV-related strategies within modern energy management frameworks, which further motivates the proposed approach.

The CDO algorithm is adopted in this study due to its superior balance between global exploration and local exploitation, making it particularly suitable for solving complex non-linear optimization problems in hybrid energy management systems, compared to conventional algorithms such as PSO, GA, and GWO.

Despite these developments, several challenges remain unaddressed in the current literature. A significant number of studies based on metaheuristic optimization^32–34^ focus primarily on offline system sizing and planning, without adequately addressing real-time operational coordination. Similarly, although AI- and DRL-based approaches^35,36^ improve system adaptability, they often introduce high computational complexity and reduced transparency, which limits their suitability for techno-economic evaluations.

Moreover, many existing works treat battery and hydrogen storage systems independently or rely on simplified rule-based coordination strategies, which fail to capture the complementary dynamics between short-term and long-term storage. This limitation restricts the ability to fully exploit system flexibility and reliability improvements.

In addition, EV integration is frequently modeled using fixed charging/discharging patterns or without considering practical constraints such as limited state-of-charge (SOC) ranges and mobility requirements. Such simplifications may lead to unrealistic system behavior and an overestimation of V2G benefits.

Furthermore, only a limited number of studies incorporate reliability indicators, such as loss of power supply probability (LPSP), directly into the optimization framework. Consequently, many existing approaches prioritize cost reduction without adequately ensuring system reliability, particularly in remote and off-grid contexts.

These limitations emphasize the need for a coordinated and constraint-aware multi-storage energy management framework that integrates BESS, HESS, and EV systems within a unified techno-economic optimization model that explicitly accounts for reliability considerations.

### Motivation and research gaps

Battery energy storage systems (BESS) are commonly employed due to their high efficiency and rapid response, which make them well-suited for short-term energy balancing. Nevertheless, their limited capability for long-duration storage constrains their effectiveness in ensuring long-term system autonomy. In contrast, hydrogen energy storage systems (HESS) offer large-scale and long-term storage potential, enabling sustained energy supply during extended deficit periods. Accordingly, combining BESS and HESS provides a complementary solution capable of addressing both short-term variability and long-term energy needs.

Meanwhile, the growing adoption of electric vehicles (EVs) introduces additional load demand alongside new operational opportunities through vehicle-to-grid (V2G) functionality. This contributes to improved system flexibility and better utilization of renewable energy resources. However, the simultaneous integration of intermittent renewable generation, hybrid storage systems, and EVs results in a complex, nonlinear, and uncertain system environment that cannot be effectively managed using conventional rule-based or single-storage energy management strategies.

Building on these identified gaps, the novelty of this study lies in:

1. The development of a coordinated multi-storage energy management strategy that integrates BESS, HESS, and constrained EV participation within a unified optimization framework.
2. The application of the CDO algorithm to solve the complex techno-economic optimization problem, providing a superior balance between exploration and exploitation compared to traditional methods.
3. A comprehensive evaluation of the economic trade-offs and reliability benefits of integrating V2G functionality in large-scale remote microgrids.

To further clarify the research gap and position the proposed work within the existing literature, a comparative summary is presented in Table 1.

**Table 1:** Comparative analysis of existing studies and identified research gaps.

Reference	Approach/System	Key Contribution	Limitations	Contribution of This Work
^20–23^	RES + storage systems	Improved economic performance and flexibility	Lack of integrated multi-storage coordination	Coordinated EMS integrating BESS, HESS, and EV
^24,32,33^	Metaheuristic optimization	Cost minimization and system sizing	Focus on offline optimization, no real-time coordination	Coordinated operational EMS within optimization
^35,36^	AI/DRL-based EMS	Adaptive and intelligent control	High complexity and low transparency	Simpler, interpretable EMS with practical constraints
^25–29^	Hybrid PV–WT–diesel–battery systems	Reduced fuel consumption and emissions	Conventional EMS, no hybrid storage coordination	Advanced multi-storage coordination strategy
^37,38^	V2G integration	Improved reliability and flexibility	Idealized EV models without constraints	Constrained EV participation (SOC + mobility limits)
Various studies	Single storage (BESS or HESS)	Improved performance individually	No interaction between short- and long-term storage	Integrated BESS–HESS coordination

### Paper contributions

While hybrid microgrid architectures and techno-economic optimization have been widely studied, the novelty of this work lies in the development of a coordinated and constraint-aware multi-storage energy management framework that integrates BESS, HESS, and EVs within a unified optimization model, leading to improved system reliability, enhanced operational flexibility, and more efficient resource utilization. The main contributions are summarized as follows:

1. A coordinated multi-storage energy management strategy (EMS) is developed for hybrid microgrids integrating BESS, HESS, and EVs, to improve system reliability and reduce overall operational cost through effective coordination between storage technologies.
2. Unlike conventional approaches, EVs are modeled as semi-available and constrained storage units with limited V2G participation to preserve mobility requirements, resulting in a more realistic and practical representation of EV integration in microgrids.
3. A unified and fair comparative optimization framework is established, enabling a reliable and unbiased techno-economic comparison between multiple system configurations evaluated under identical constraints and bounds.
4. The study provides a detailed analysis of the interaction between different storage technologies, demonstrating improved system efficiency through reduced battery oversizing and limited reliance on hydrogen systems.
5. The proposed framework integrates reliability constraints (LPSP) directly within the optimization process, ensuring a balanced trade-off between cost, reliability, and energy utilization.

## Methods

The implementation of electrification in rural and isolated locations presents a major technical and economic challenge. Addressing this issue requires considering the challenges that isolated places may encounter, for example, the high costs of infrastructure, geographical constraints, as well as limited supply of conventional energy.

The following section details the proposed microgrid architecture for supplying electricity to an off-grid community in Egypt.

### Methodology

Providing reliable electricity access in rural areas remains a critical challenge.

The proposed optimization framework follows a structured three-stage process:

(1) System Inputs renewable energy resource data (hourly solar irradiance, ambient temperature, and wind speed) obtained from NASA POWER for the selected Egyptian site, along with hourly load demand profiles and component technical/economic parameters (Table 7).

(2) Optimization Problem Formulation the decision variables are the number of PV units (N_PV), wind turbines (N_WT), battery units (N_batt), hydrogen storage units (N_H2), and EV units (N_EV), and the objective is to minimize the 20-year Net Present Cost (NPC) subject to a reliability constraint (LPSP ≤ ε) enforced through a penalty function.

(3) Outputs optimal component sizing, total NPC, LPSP, and hourly energy dispatch profiles for each storage technology.

The energy management priority during surplus generation follows the sequence:

(i) charge EVB to target SOC, (ii) charge BESS, (iii) produce hydrogen via electrolyzer (HESS charging), and (iv) direct excess to dummy load.

During deficit conditions, the discharge sequence is: (i) BESS discharge, (ii) HESS fuel cell output, (iii) EVB discharge (limited to 10% of usable capacity to preserve mobility).

This deterministic rule-based EMS is embedded within the optimization loop and applied consistently across all 8,760 annual hourly time steps.

These regions commonly face considerable challenges, including high infrastructure costs, geographical constraints, and limited access to conventional energy resources. Accordingly, the selection of appropriate electrification solutions is critical. Renewable energy–based off-grid microgrids offer a cost-effective and sustainable alternative for electrifying remote areas. This study provides a comprehensive evaluation of electricity supply for a remote site in Cairo, located in the northern region of Egypt. The generation technologies are selected based on the site’s geographical and resource characteristics. Two primary renewable sources are considered: photovoltaic (PV) generation and wind turbines (WTs). Because renewable energy sources are intermittent, the microgrid needs additional backup and energy storage systems. The proposed backup/storage system includes a battery energy storage system (BESS), a hydrogen energy storage unit (HESS), and batteries from electric vehicles (EVs). The technical specifications of the proposed microgrid components are summarized in Table 7. This paper proposes a structured method for optimizing the design and operation of an isolated microgrid supported by a dependable hybrid backup system. The model aims to optimize microgrid sizing and operation for areas lacking reliable utility-grid access, such as remote or off-grid locations. A robust backup system is essential for maintaining supply continuity and enhancing microgrid resilience under adverse conditions. Accordingly, a hybrid backup configuration is presented using three energy storage sources including BESS, HESS, and EV, with the objective of determining an optimal system design consistent with Egypt’s climatic and operational conditions. BESS is widely used for short-term backup, whereas HESS enables long-term, low-carbon energy storage by converting electricity to hydrogen and reconverting it to electricity when needed. The third source is the EVB, which can support low-carbon operation through vehicle-to-grid (V2G) participation when properly coordinated. EVs owners can benefit by exporting energy to the grid during peak-demand periods and recharging during off-peak hours, subject to availability and SOC constraints.

The model aims to maximize the utilization of renewable energy sources (RESs) and manage storage dispatch and demand operational strategies that maintain stable and sustainable microgrid performance. This approach integrates RES-based generation with hybrid backup/storage systems to ensure reliable and efficient power supply.

This study adopts a deterministic modeling framework to enable a clear and consistent techno-economic evaluation of different system configurations under identical operating conditions. Although uncertainty in renewable generation, load demand, and market conditions is recognized as an important factor in real-world systems, it is not explicitly considered in the current model to maintain clarity and tractability. Incorporating stochastic or robust optimization approaches is considered a valuable direction for future work.

In addition, the simulation setup is designed to reflect realistic operating conditions. The selected parameter values, including component efficiencies, capacity limits, and cost coefficients, are based on commonly reported data in the literature and representative system characteristics.

### Modeling of the hybrid energy system

**Fig. 1:**
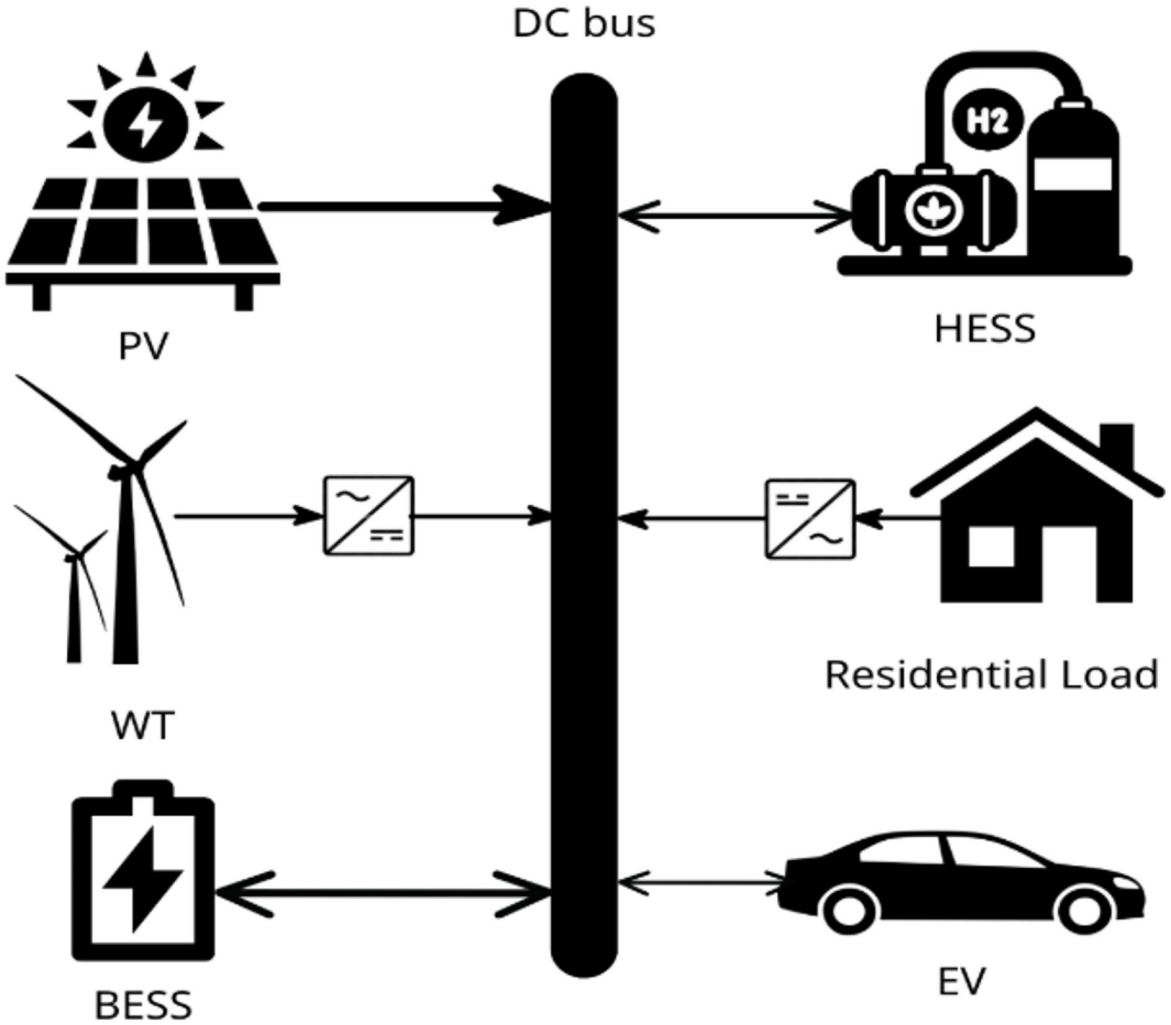
Configuration of the BESS/HESS/EVB-based backup system.

The mathematical formulation of the proposed hybrid renewable system is presented in this section, incorporating PV arrays, wind turbines, batteries, electric vehicles, fuel cells, an electrolyzer, a hydrogen storage tank, and an inverter. Figure 1 illustrates the system configuration. The analysis considers an off-grid system supplying electricity to remote loads, where PV and WT are connected to a DC bus and feed the AC load through an inverter.

Surplus PV/WT power is allocated to charge the BESS, produce hydrogen via the electrolyzer (HESS charging), and charge the EVB; the fuel cell supplies power during deficits. In the event of insufficient PV/WT generation, the BESS, HESS, and EVB are connected to the DC bus.

The mathematical models used in this study are based on standard formulations reported in the literature, with appropriate references provided. Some equations have been adapted and integrated within the proposed framework to suit the specific system configuration.

All variables, parameters, and indices used in the mathematical formulation are consistently defined and summarized in Table 2 to improve clarity and reproducibility of the proposed model.

**Table 2:** Definition of variables and parameters used in the mathematical model.

Symbol	Description	Unit
$\:\boldsymbol{S}{\boldsymbol{R}}_{\boldsymbol{i}\boldsymbol{n}\boldsymbol{t}}\left(\boldsymbol{t}\right)$	Solar irradiance at time t	W/m²
$\:\boldsymbol{P}{\boldsymbol{V}}_{\boldsymbol{N}}$	Number of PV units	–
$\:\boldsymbol{P}{\boldsymbol{V}}_{\boldsymbol{P}}^{\boldsymbol{r}\boldsymbol{a}\boldsymbol{t}}$	Rated power of PV unit	kW
$\:{\boldsymbol{W}}_{\boldsymbol{\eta\:}}$	Wiring efficiency	–
$\:\boldsymbol{P}{\boldsymbol{V}}_{\boldsymbol{\eta\:}}$	PV module efficiency	–
$\:{\boldsymbol{\delta\:}}_{\boldsymbol{T}}$	Temperature coefficient of PV maximum power	1/°C
$\:{\boldsymbol{T}}_{\boldsymbol{A}}$	Ambient temperature	°C
$\:{\boldsymbol{T}}_{\boldsymbol{r}}$	Reference temperature	°C
$\:{\boldsymbol{T}}_{\boldsymbol{n}\boldsymbol{o}\boldsymbol{r}}$	Nominal cell temperature	°C
$\:{\mathbf{N}}_{\mathbf{W}\mathbf{T}}$	Number of wind turbines	–
$\:{\boldsymbol{\eta\:}}_{\boldsymbol{W}\boldsymbol{T}}$	Wind turbine efficiency	–
$\:{\boldsymbol{P}}_{\boldsymbol{W}\boldsymbol{T}}^{\boldsymbol{r}\boldsymbol{a}\boldsymbol{t}\boldsymbol{e}\boldsymbol{d}}$	Rated power of wind turbine	kW
$\:{\boldsymbol{v}}_{\boldsymbol{c}\boldsymbol{u}\boldsymbol{t}\mathrm{-}\boldsymbol{i}\boldsymbol{n}}$	Cut-in wind speed	m/s
$\:{\boldsymbol{v}}_{\boldsymbol{c}\boldsymbol{u}\boldsymbol{t}\mathrm{-}\boldsymbol{o}\boldsymbol{f}\boldsymbol{f}}$	Cut-off wind speed	m/s
Σ	Battery self-discharge rate	1/h
$\:{\boldsymbol{E}}_{\boldsymbol{G}\boldsymbol{e}\boldsymbol{n}}\left(\boldsymbol{t}\right)$	Generated energy from RES	kW
$\:{\boldsymbol{E}}_{\boldsymbol{L}\boldsymbol{o}\boldsymbol{a}\boldsymbol{d}}\left(\boldsymbol{t}\right)$	Load demand	kW
$\:{\boldsymbol{\eta\:}}_{\boldsymbol{i}\boldsymbol{n}\boldsymbol{v}}$	Inverter efficiency	–
$\:{\boldsymbol{\eta\:}}_{\boldsymbol{B}\boldsymbol{a}\boldsymbol{t}\boldsymbol{t}}$	Battery charging efficiency	–
$\:{\boldsymbol{E}}_{\boldsymbol{b}\boldsymbol{a}\boldsymbol{t}\boldsymbol{t},\boldsymbol{m}\boldsymbol{i}\boldsymbol{n}}$	Minimum battery energy	kWh
$\:{\boldsymbol{E}}_{\boldsymbol{b}\boldsymbol{a}\boldsymbol{t}\boldsymbol{t},\boldsymbol{m}\boldsymbol{a}\boldsymbol{x}}$	Maximum battery energy	kWh
$\:{\boldsymbol{D}\boldsymbol{O}\boldsymbol{D}}_{\boldsymbol{m}\boldsymbol{a}\boldsymbol{x}}$	Maximum depth of discharge	–
C_B_	Battery capacity	kWh
$\:{\boldsymbol{P}}_{\boldsymbol{e}\boldsymbol{l}\boldsymbol{e}-{\boldsymbol{H}}_{2}\boldsymbol{t}}$	Electrolyzer output power	kW
$\:{\boldsymbol{P}}_{\boldsymbol{r}\boldsymbol{e}\boldsymbol{n}-\boldsymbol{e}\boldsymbol{l}\boldsymbol{e}}$	Electrolyzer input power	kW
$\:{\boldsymbol{\eta\:}}_{\boldsymbol{e}\boldsymbol{l}\boldsymbol{e}}$	Electrolyzer efficiency	–
$\:{\boldsymbol{m}}_{{\boldsymbol{H}}_{2}}$	Hydrogen mass produced	kg
$\:\boldsymbol{E}\boldsymbol{L}$	Electrolysis factor	–
$\:{\boldsymbol{M}}_{\boldsymbol{H}\boldsymbol{T}}$	Margin coefficient	–
$\:{\boldsymbol{P}}_{{\boldsymbol{H}}_{2}}$	Hydrogen pressure	bar
$\:{\boldsymbol{\rho\:}}_{{\boldsymbol{H}}_{2}}$	Hydrogen density	kg/m³
$\:{\boldsymbol{E}}_{\boldsymbol{R}\boldsymbol{E}\boldsymbol{S}}\left(\boldsymbol{t}\right)$	Renewable energy generation	kW
$\:{\boldsymbol{\eta\:}}_{\boldsymbol{F}\boldsymbol{C}\boldsymbol{s}}$	Fuel cell efficiency	–
$\:{\boldsymbol{V}}_{\boldsymbol{F}\boldsymbol{C}}$	Fuel cell voltage	V
$\:{\boldsymbol{V}}_{\boldsymbol{a}\boldsymbol{c}\boldsymbol{t}}$	Activation loss voltage	V
$\:{\boldsymbol{V}}_{\boldsymbol{c}\boldsymbol{o}\boldsymbol{n}}$	Concentration loss voltage	V
$\:{\boldsymbol{V}}_{\boldsymbol{o}\boldsymbol{h}\boldsymbol{m}}$	Ohmic loss voltage	V
$\:{\boldsymbol{E}}_{\boldsymbol{N}\boldsymbol{e}\boldsymbol{r}\boldsymbol{n}\boldsymbol{s}\boldsymbol{t}}$	Nernst voltage	V
T	Temperature	K
$\:{\boldsymbol{P}}_{{\boldsymbol{O}}_{2}}$	Oxygen pressure	atm
$\:{\boldsymbol{\epsilon\:}}_{1},{\boldsymbol{\epsilon\:}}_{2},{\boldsymbol{\epsilon\:}}_{3},{\boldsymbol{\epsilon\:}}_{4}$	Fuel cell empirical coefficients	–
$\:{\boldsymbol{I}}_{\boldsymbol{F}\boldsymbol{C}}$	Fuel cell current	A
$\:\boldsymbol{n}$	Number of electrons	–
$\:\boldsymbol{R}3$	Universal gas constant	J/mol·K
$\:\boldsymbol{F}$	Faraday constant	C/mol
$\:{\boldsymbol{i}}_{1}$	Limiting current density	A/m²
$\:{\boldsymbol{R}}_{\boldsymbol{c}}$	Conduction resistance	Ω
$\:{\boldsymbol{R}}_{\boldsymbol{m}}$	Membrane resistance	Ω
$\:{\boldsymbol{n}}_{\boldsymbol{e}\boldsymbol{x}}$	Number of electrons transferred	–
$\:{\boldsymbol{U}}_{\boldsymbol{F}}$	Fuel utilization factor	–
$\:{\boldsymbol{m}}_{\boldsymbol{c}\boldsymbol{o}\boldsymbol{m}\boldsymbol{b}}$	Mass flow rate	kg/s
$\:{\boldsymbol{P}}_{\boldsymbol{F}\boldsymbol{C}}$	Fuel cell output power	kW
$\:{\boldsymbol{Q}}_{\boldsymbol{L}\boldsymbol{H}\boldsymbol{V}}$	Lower heating value	kJ/mol
$\:\boldsymbol{S}\boldsymbol{O}{\boldsymbol{C}}_{\boldsymbol{i}\boldsymbol{n}\boldsymbol{t}}$	Initial state of charge	–
$\:\boldsymbol{C}\boldsymbol{A}{\boldsymbol{P}}_{\boldsymbol{E}\boldsymbol{V}}^{\boldsymbol{f}\boldsymbol{u}\boldsymbol{l}\boldsymbol{l}}$	EV battery capacity	kWh
$\:\boldsymbol{\Delta\:}{\boldsymbol{P}}_{\boldsymbol{r}\boldsymbol{G}2\boldsymbol{V}}$	Grid-to-vehicle charging power	kW
$\:\boldsymbol{\Delta\:}{\boldsymbol{P}}_{\boldsymbol{r}\boldsymbol{V}2\boldsymbol{H}}$	Vehicle-to-home power	kW
$\:\boldsymbol{\Delta\:}{\boldsymbol{P}}_{\boldsymbol{r}\boldsymbol{V}2\boldsymbol{G}}$	Vehicle-to-grid power	kW
$\:{\boldsymbol{P}}_{\boldsymbol{r}\boldsymbol{E}\boldsymbol{V},\boldsymbol{m}\boldsymbol{i}\boldsymbol{n}}$	Minimum EV power	kW
$\:{\boldsymbol{P}}_{\boldsymbol{r}\boldsymbol{E}\boldsymbol{V},\boldsymbol{m}\boldsymbol{a}\boldsymbol{x}}$	Maximum EV power	kW
$\:\boldsymbol{S}\boldsymbol{O}{\boldsymbol{C}}_{\boldsymbol{E}\boldsymbol{V},\boldsymbol{m}\boldsymbol{i}\boldsymbol{n}}$	Minimum EV SOC	–
$\:\boldsymbol{S}\boldsymbol{O}{\boldsymbol{C}}_{\boldsymbol{E}\boldsymbol{V},\boldsymbol{m}\boldsymbol{a}\boldsymbol{x}}$	Maximum EV SOC	–

#### Solar photovoltaic (PV) module

PV modules employ the photovoltaic effect to convert solar irradiance into electricity, providing a clean energy source^58^. The PV array output power ($\:{PV}_{P}$) and cell temperature ($\:{T}_{c}$) may be calculated using the formulae below^59^.

1 $$\:{PV}_{P}\left(t\right)=\left(\frac{{SR}_{int}\left(t\right)}{1000}\right)\left({PV}_{N}{PV}_{P}^{rat}{{W}_{\eta\:}PV}_{\eta\:}\right)(1-{\delta\:}_{T}\left({T}_{c}-{T}_{r}\right))$$

2 $$\:{T}_{c}\left(t\right)={SR}_{int}\left(t\right)\left(\frac{{T}_{nor}-20}{800}\right)+{T}_{A}$$

The variables $\:S{R}_{int}\left(t\right)$, $\:P{V}_{N}$, $\:P{V}_{P}^{rat}$, $\:{W}_{\eta\:}$, and $\:P{V}_{\eta\:}$correspond to the solar irradiance at time *\:t*, the number of PV units, the rated power, the wiring efficiency, and the PV module efficiency, respectively.

The temperature coefficient of maximum power ($\:{\delta\:}_{T}$), ambient temperature ($\:{T}_{A}$), and reference temperature ($\:{T}_{r}$) for the PV module are all set to 25 °C. The nominal cell temperature ($\:{T}_{nor}$) is defined under standard operating conditions, based on 800 W/m² solar radiation, 1 m/s wind speed, 20 °C ambient temperature, and a 45° tilt angle.

#### Wind farm modelling

The power output of a wind farm at a given wind speed $\:v\left(t\right)$can be analytically modeled based on aerodynamic and wind energy principles^60^.

3 $$\:PWT\left(t\right)=\left\{\begin{array}{c}\:\:\:\:0\:\:\:\:\:\:\:\:\:\:\:\:\:\:\:\:\:\:\:\:\:\:\:\:\:\:\:\:\:\:\:\:\:\:\:\:\:\:\:\:\:\:\:\:\:\:\:\:\:\:\:\:\:\:\:\:\:\:\:\:\:\:\:\:\:\:\:\:\:\:\:{\:\:\:\:\:\:\:\:\:v}_{cut-in}\:\:\:\:\le\:\:\:\:\:v\left(t\right)\:<\:\:{v}_{cut-off}\\\:\frac{{N}_{WT}{\eta\:}_{WT}{P}_{WT\_rated}\left({v}^{2}\right(t)-\:{v}_{cut-in}^{2})}{({v}_{rated}^{2}-{v}_{cut-in}^{2})}\:\:\:\:\:\:\:\:\:\:\:\:\:\:\:\:\:\:\:\:\:\:\:\:{v}_{cut-in}\:\le\:v\left(t\right)\:<{v}_{rated}\:\:\\\:\:{N}_{WT}{\eta\:}_{WT}{P}_{WT\_rated}\:\:\:\:\:\:\:\:\:\:\:\:\:\:\:\:\:\:\:\:\:\:\:\:\:\:\:\:\:\:\:\:\:\:\:\:\:\:\:\:\:\:\:\:\:\:\:\:\:{v}_{rated}\:\:\le\:v\left(t\right)\:<{v}_{cut-off}\:\:\:\:\end{array}\right.$$

$\:{\mathrm{N}}_{\mathrm{W}\mathrm{T}}$ denotes the number of wind turbines within the farm, $\:{\eta\:}_{WT}$represents the overall efficiency, $\:{P}_{WT}^{rated}$is the nominal power output at rated wind speed, and $\:{v}_{cut\mathrm{-}in}$and $\:{v}_{cut\mathrm{-}off}$correspond to the cut-in and cut-off wind speeds at which the turbine initiates generation and ceases operation for safety, respectively.

#### Backup system modeling

Reliable backup energy sources are fundamental for maintaining the stability and dependability of electrical power systems, where the balance between generation and demand represents a complex and dynamic operation. Backup energy sources are essential for ensuring system stability and flexibility under varying demand conditions. The proposed system integrates BESS, HESS, and EVB as backup and energy storage components. BESS operates as a backup power source, whereas HESS provides an environmentally sustainable solution by generating electricity from hydrogen. The third source, EVB, offers benefits for both the microgrid and EV owners. Through vehicle-to-grid (V2G) functionality, EVs can supply energy during peak demand periods, subject to availability and SOC constraints. Coordinated EVB operation can reduce peak stress by shifting charging to off-peak periods and enabling controlled discharging during peak demand. The following subsections provide detailed mathematical modeling of each backup energy source considered in this study.

##### Battery energy storage system (BESS)

Maintaining equilibrium between electricity generation and demand is crucial for ensuring the reliability of power systems. The integration of renewable energy sources (RESs) introduces challenges due to their inherent intermittency. Even minor factors, such as transient cloud cover and wind speed variability, can significantly reduce power generation. Maintaining grid stability gets increasingly difficult as supply and demand changes rise. Electricity demand is influenced by diurnal patterns, seasonal variations, and consumer consumption behavior, while BESSs offer an efficient means of addressing challenges in smart grid operation. Energy storage systems enhance the stability of electricity generation and ensure demand satisfaction through the storage of surplus energy and its subsequent delivery to the grid when required, with ESSs anticipated to act as key power sources in future smart grids. BESSs are charged using excess electricity generated from renewable sources during periods of strong wind or high solar irradiance. During changes in supply or demand, power can be injected back into the grid to stabilize the system, during surplus generation, the batteries are charged.

The state of charge (SOC) of a battery is crucial for determining its performance and capacity.

The SOC may be determined based on both charging and discharging states^61,62^.

Where:

4 $$\:\left({P}_{PV}\left(t\right)+{P}_{WT}\left(t\right)\right)dt={E}_{Gen}\left(t\right)$$

$$\:\left({P}_{Load}\left(t\right)\right)dt={E}_{Load}\left(t\right)$$

Charging process, if; $\:{{E}_{Gen}\left(t\right)>E}_{Load}\left(t\right)$

5 $$\:{E}_{Batt}\left(t\right)={E}_{Batt}\left(t-1\right)\left(1-\sigma\:\right)+\left({E}_{Gen}\left(t\right)-\frac{{E}_{Load}\left(t\right)}{{\eta\:}_{inv}}\right){\eta\:}_{Batt}$$

Discharging process, if; $\:{{E}_{Gen}\left(t\right)<E}_{Load}\left(t\right)$

6 $$\:{E}_{Batt}\left(t\right)={E}_{Batt}\left(t-1\right)\left(1-\sigma\:\right)-\left(\frac{{E}_{Load}\left(t\right)}{{\eta\:}_{inv}}-{E}_{Gen}\left(t\right)\right){\eta\:}_{Batt}$$

The energy stored in the battery system is constrained by the following conditions:

$$\:{E}_{batt,min}\left(t\right)\le\:{E}_{batt}\left(t\right)\le\:{E}_{batt,max}\left(t\right)$$

Where,

7 $$\:{E}_{batt,max}{=SOC}_{max}{C}_{B}$$

8 $$\:{E}_{batt,min}=(1-{DOD}_{max}){C}_{B}$$

In this equation, σ denotes the hourly self-discharge rate, $\:{E}_{Gen}\left(t\right)$ represents the produced power from renewable energy sources, and $\:{E}_{Load}\left(t\right)$ represents the load demand, $\:{\eta\:}_{inv}$ (inverter efficiency), $\:{\eta\:}_{Batt}$ (charge efficiency factor), $\:{E}_{batt,min}\&{E}_{batt,max}$ (minimum and maximum energy stored), $\:{DOD}_{max}$ (the maximum allowable depth to which the battery can be discharged), C_B_ (battery’s rated energy capacity).

##### Hydrogen energy storage system technology

HESS is recognized as a promising energy storage technology that enables the storage of surplus energy as hydrogen gas. The major purpose is to handle the intermittent nature of renewable energy sources like solar and wind power. HESS converts extra power into hydrogen, providing energy storage for subsequent utilization. The HESS comprises an electrolyzer, a hydrogen storage tank, fuel cells, and a local microcontroller. The process is initiated by the electrolyzer, which performs water electrolysis to produce hydrogen, and the generated hydrogen is subsequently stored in specially designed tanks. A local microprocessor controls hydrogen generation and conversion to power. The microprocessor manages the system’s phases, ensuring efficient and seamless operation from hydrogen production to energy conversion through fuel cells (FCs).

- Electrolyzer.

Electrolyzers utilize electrical energy to split water into hydrogen and oxygen, with individual cells connected in series. The amount of hydrogen produced can be determined by evaluating the energy required to generate 1 kg of hydrogen. The mathematical expression for hydrogen production by the electrolyzer is given as follows^63^:

9 $$\:{Electricity+H}_{2}O={H}_{2}+\frac{1}{2}{O}_{2}$$

The power flow from the electrolyzer to the hydrogen storage system is defined by the following equation^63^:

10 $$\:{P}_{ele-{H}_{2}t}={P}_{ren-ele}\times\:{\eta\:}_{ele}$$

where $\:{P}_{ele-{H}_{2}t}$ is the electrolyzer output power (kw), $\:{P}_{ren-ele}$ is the electrolyzer input power (kw), and $\:{\eta\:}_{ele}$ is the electrolyzer efficiency (a constant value).

- Hydrogen tanks.

Hydrogen tanks are engineered to securely and efficiently store hydrogen gas, enabling its use in various applications such as power generation and industrial activities. Hydrogen is typically produced and stored at elevated pressures (e.g., ~ 30 bar) and then regulated to the fuel cell operating pressure (e.g., 1–2 bar) to ensure safe and reliable performance. The storage capacity of the hydrogen tank can be evaluated using the following equation^63^:

11 $$\:{V}_{{H}_{2}}=\frac{{m}_{{H}_{2}}\times\:EL\times\:{M}_{HT}}{{P}_{{H}_{2}}\times\:{\rho\:}_{{H}_{2}}}$$

where, $\:{m}_{{H}_{2}}\times\:EL$ corresponds to the hydrogen produced by the electrolyzer, $\:{M}_{HT}$is the margin coefficient, $\:{P}_{{H}_{2}}$denotes the hydrogen pressure, and $\:{\rho\:}_{{H}_{2}}$is the hydrogen density (0.089 kg/m³). The hydrogen quantity stored in the tank during the electrolyzer charging phase is computed using Eq. (12)^60^, whereas during the discharging phase, when fuel cells (FCs) consume hydrogen to mitigate power shortages, the stored hydrogen is evaluated using Eq. (13):

12 $$\:\:{E}_{stored}\left(t\right)={E}_{stored}\left(t-1\right)+\left[{E}_{RES}\left(t\right)-\frac{{E}_{load}\left(t\right)}{{\eta\:}_{inv}}\right]\times\:{\eta\:}_{ELE}$$

13 $$\:{E}_{stored}\left(t\right)={E}_{stored}\left(t-1\right)-\frac{\left[\frac{{E}_{load}\left(t\right)}{{\eta\:}_{inv}}-{E}_{RES}\left(t\right)\right]}{{\eta\:}_{FCs}\times\:{\eta\:}_{inv}}$$

where, $\:{E}_{RES}\left(t\right)$ represents the power generated from renewable sources, $\:{E}_{load}\left(t\right)$denotes the load demand, while $\:{\eta\:}_{inv}$and $\:{\eta\:}_{FCs}$correspond to the efficiencies of the inverter and fuel cells, respectively.

- Fuel cell technology.

During periods of increased demand or reduced renewable generation, stored hydrogen is utilized for electricity production. A proton-exchange membrane fuel cell (PEMFC) is adopted in this study, as it represents one of the most widely used fuel cell technologies.

The PEMFC technology is adopted due to its fast dynamic response, high efficiency, and suitability for microgrid applications with variable renewable energy generation.

The fuel cell output voltage ($\:{V}_{FC}$) is influenced by activation, concentration, and ohmic losses ($\:{V}_{act}$, $\:{V}_{con}$, and $\:{V}_{ohm}$), as formulated in Eqs. (16), (18), (20), and (22)^64^:

14 $$\:{V}_{FC}={E}_{Nernst}-{V}_{act}-{V}_{con}-{V}_{ohm}$$

where, $\:{E}_{Nernst}$ denotes the voltage generated as a result of the electrochemical reaction, as illustrated below.

15 $$\:\:{E}_{Nernst}=1.229-8.46\times\:{10}^{-4}\left(T-298.15\right)+4.31\times\:\:{10}^{-5}T(\mathrm{ln}({P}_{{H}_{2}}\times\:{P}_{{O}_{2}}^{\frac{1}{2}})$$

where, T and $\:{P}_{{O}_{2}}$are the temperature and oxygen pressure, respectively.

16 $$\:\:\:{V}_{act}=-\left[{\epsilon\:}_{1}+{\epsilon\:}_{2}T+{\epsilon\:}_{3}T\mathrm{ln}\left({CO}_{2}\right)+{\epsilon\:}_{4}T\mathrm{ln}\left({I}_{FC}\right)\right]$$

Here, $\:{\epsilon\:}_{1},{\epsilon\:}_{2},{\epsilon\:}_{3},{\epsilon\:}_{4}$are fuel-cell parameters, and $\:{I}_{FC}$denotes the fuel-cell current.

2 is the oxygen concentration defined as:

17 $$\:\:\:{CO}_{2}=\frac{{P}_{{O}_{2}}}{5.08\times\:{10}^{6}\:\times\:{exp}^{\left(\frac{-498}{T}\right)}}$$

18 $$\:\:\:{V}_{con}=\frac{RT}{nF}.\mathrm{ln}\left(1-\frac{{I}_{FC}}{{i}_{1}}\right)$$

where *\:n* denotes the number of electrons, *\:R* and *\:F* represent the ideal gas constant and Faraday constant, respectively, and $\:{i}_{1}$corresponds to the limiting current density.

19 $$\:{V}_{ohm}={I}_{FC}\times\:\left({R}_{m}+{R}_{c}\right)$$

where $\:{R}_{c}$ represents the conduction resistance and $\:{R}_{m}$ corresponds to the membrane resistance.

20 $$\:{I}_{FC}={n}_{ex}\times\:F\times\:{U}_{F}\times\:{\dot{m}}_{comb}$$

where, $\:{n}_{ex}$, $\:{U}_{F}$, and $\:{m}_{comb}$ denote the number of electrons transferred, the fuel utilization factor, and the mass flow rate (kg·s⁻¹), respectively.

The following equations are used to evaluate the output power ($\:{P}_{FC}$) and efficiency ($\:{\eta\:}_{FC}$) of the fuel cells:

21 $$\:{P}_{FC}={{V}_{FC}\times\:I}_{FC}$$

22 $$\:{\eta\:}_{FC}=\frac{{{V}_{FC}\times\:I}_{FC}}{{Q}_{LHV}\times\:{U}_{F}\times\:{\dot{m}}_{comb}}$$

where $\:{Q}_{LHV}$ indicates the lower heating value (KJ. mol^− 1^).

##### Electric vehicle SOC and availability modeling

The energy contribution of the EVB to supplying the grid or household is constrained by the following equation^65^:

23 $$\:{SOC}_{EV}={SOC}_{int}+\frac{\varDelta\:{P}_{rG2V}+\varDelta\:{P}_{rV2H}+\varDelta\:{P}_{rV2G}}{{CAP}_{EV}^{full}}$$

$$\begin{gathered} \:{P_{rEV,\max }} \geqslant \:{P_{rdis}} \hfill \\ \:{P_{rch}} \geqslant \:{P_{rEV,\min }} \hfill \\ \end{gathered}$$

To extend the lifespan of the EV battery, it should be safeguarded against overcharging and deep discharging.

$$\:{SOC}_{EV,min}\le\:{SOC}_{EV}\le\:{SOC}_{EV,max}$$

The state of the EV can be expressed by the following equation:

$$\:{LOC}_{EV}=\left\{\begin{array}{c}1\:EV\:available\\\:0\:EV\:notavailable\end{array}\right.$$

where $\:SO{C}_{int}$ denotes the initial state of charge, $\:CA{P}_{EV}^{full}$ represents the full capacity of the EV battery, $\:{\Delta\:}{P}_{rG2V}$ is the grid-to-vehicle charging power, $\:{\Delta\:}{P}_{rV2H}$ indicates the power supplied to the home, and $\:{\Delta\:}{P}_{rV2G}$ represents the power delivered to the grid. Additionally, $\:{P}_{rEV,min}$ and $\:{P}_{rEV,max}$ define the minimum and maximum operating power of the EV, respectively, while $\:SO{C}_{EV,min}$and $\:SO{C}_{EV,max}$ denote the minimum and maximum state of charge limits of the EV battery.

The EV availability parameter (LOC_EV) is defined using a deterministic schedule based on typical daily usage patterns. EVs are assumed to be connected to the microgrid during specific time intervals (e.g., evening and nighttime hours) and disconnected during mobility periods (e.g., daytime hours).

A total of approximately 80,000 EV units are considered in the system, representing aggregated EV participation in the large-scale microgrid.

### Overview of the proposed hybrid backup system

Backup energy sources are essential in stand-alone microgrids to ensure supply continuity during renewable-generation shortages or contingency conditions.

These sources act as auxiliary energy systems, supplying power when the primary generation is insufficient or unavailable, thereby enhancing the reliability and stability of microgrid operation. The system configurations are based on HESS, BESS, and EVB. As illustrated in Fig. 1, the initial microgrid configuration incorporates a hybrid backup system consisting of BESS, EVB, and HESS, where all three sources can be utilized to compensate for power deficits. The selection of the most appropriate source is determined by evaluating their operational states, including hydrogen storage levels and the state of charge (SOC) of both BESS and EVB. Moreover, surplus energy is managed according to the condition of these sources, with EVB charging prioritized during excess generation depending on availability constraints and the adopted EMS strategy.

In situations where backup sources cannot cover the energy demand, microgrid operators may apply corrective actions such as load shedding, which involves disconnecting non-essential loads to preserve supply to critical ones. Although increasing backup sources raises system costs, the EMS optimizes operation by selecting the most economical sources. Its decision-making process considers operating costs, real-time pricing, and the status of available backup resources.

### Objective function and system constraints

Designing an efficient and dependable microgrid requires careful consideration of several parameters. Priority should be given to technical and economic criteria. In this work, a complete model is developed. The suggested microgrid is optimized for efficiency by considering both technical and economic parameters.

#### Economic criteria

Microgrids’ performance and long-term viability rely heavily on their economic aspects, particularly their running costs. This study aims to improve performance and sustainability by optimizing operational decisions and O&M expenditures. Operation and maintenance (O&M) costs in microgrids are incurred to maintain system efficiency and ensure that all equipment operates under optimal condition. Maintaining optimal energy output requires daily repairs and preventative maintenance charges throughout the plant’s lifespan.

The total system NPC is categorized into capital cost, replacement cost, and O&M cost. The cost of each component is calculated using the following expressions^66^:

24 $$\:{{\mathrm{C}}_{\mathrm{t}}^{\mathrm{P}\mathrm{V}}=N}_{PV}\left({C}_{PV}^{C}+\left({C}_{PV}^{M}\left(\frac{{\left(1+i\right)}^{n}-1}{{i\left(1+i\right)}^{n}}\right)\right)\right)$$

25 $$\:{{\mathrm{C}}_{\mathrm{t}}^{\mathrm{W}}=N}_{W}\left({C}_{W}^{C}+\left({C}_{W}^{M}\left(\frac{{\left(1+i\right)}^{n}-1}{{i\left(1+i\right)}^{n}}\right)\right)\right)$$

26 $$\:{{\mathrm{C}}_{\mathrm{t}}^{\mathrm{F}\mathrm{C}}=N}_{FC}\left({C}_{FC}^{C}+{C}_{FC}^{R}\sum\:_{\mathrm{j}=1}^{\mathrm{j}=(\frac{\mathrm{n}}{\mathrm{n}\mathrm{F}\mathrm{C}}-1)}\left(1+\frac{1}{{\left(1+i\right)}^{jnFC}}\right)+\left({C}_{FC}^{M}\left(\frac{{\left(1+i\right)}^{n}-1}{{i\left(1+i\right)}^{n}}\right)\right)\right)$$

27 $$\:{{\mathrm{C}}_{\mathrm{t}}^{\mathrm{E}\mathrm{l}\mathrm{e}\mathrm{c}}=N}_{Elec}\left({C}_{Elec}^{C}+{C}_{Elec}^{R}\sum\:_{\mathrm{j}=1}^{\mathrm{j}=(\frac{\mathrm{n}}{\mathrm{n}\mathrm{E}\mathrm{l}\mathrm{e}\mathrm{c}}-1)}\left(1+\frac{1}{{\left(1+i\right)}^{jnElec}}\right)+\left({C}_{Elec}^{M}\left(\frac{{\left(1+i\right)}^{n}-1}{{i\left(1+i\right)}^{n}}\right)\right)\right)$$

28 $$\:{{\mathrm{C}}_{\mathrm{t}}^{\mathrm{T}\mathrm{a}\mathrm{n}\mathrm{k}}=N}_{Tank}\left({C}_{Tank}^{C}+\left({C}_{Tank}^{M}\left(\frac{{\left(1+i\right)}^{n}-1}{{i\left(1+i\right)}^{n}}\right)\right)\right)$$

29 $$\:{{\mathrm{C}}_{\mathrm{t}}^{\mathrm{B}\mathrm{a}\mathrm{t}\mathrm{t}}=N}_{Batt}{E}_{Batt-rated}\left({C}_{Batt}^{C}+{C}_{Batt}^{R}\sum\:_{\mathrm{j}=1}^{\mathrm{j}=(\frac{\mathrm{n}}{\mathrm{n}\mathrm{B}\mathrm{a}\mathrm{t}\mathrm{t}}-1)}\left(1+\frac{1}{{\left(1+i\right)}^{jnBatt}}\right)+\left({C}_{Batt}^{M}\left(\frac{{\left(1+i\right)}^{n}-1}{{i\left(1+i\right)}^{n}}\right)\right)\right)\:\:\:\:\:\:\:\:\:\:\:$$

The O&M cost of the BESS is assumed to be relatively low, consistent with literature values typically reported in the range of 1–3% of the capital cost per year for lithium-ion battery systems.

30 $$\:{{\mathrm{C}}_{\mathrm{t}}^{\mathrm{E}\mathrm{V}}=N}_{EV}{E}_{EV-rated}{C}_{EV}^{C}$$

31 $$\:{{\mathrm{C}}_{\mathrm{t}}^{\mathrm{I}\mathrm{n}\mathrm{v}}=N}_{Inv}{C}_{Inv}^{C}$$

The objective function, representing the net present cost (NPC), can be written as follows:

32 $$\:{NPC=C}_{t}^{PV}+{C}_{t}^{W}+{C}_{t}^{FC}+{C}_{t}^{Elec}+{C}_{t}^{Tank}+{C}_{t}^{Batt}+{C}_{t}^{EV}+{C}_{t}^{Inv}$$

 

It should be noted that the salvage value of system components at the end of the project lifetime is not explicitly considered in the NPC calculation. This assumption is adopted for model simplification, and its impact is expected to be relatively limited compared to capital and replacement costs.

#### Reliability metric

When sizing microgrid components, it’s crucial to prioritize dependability. This study uses the LPSP, a well-established metric, to assess system dependability. The unpredictable nature of renewable energy sources (RESs) poses a significant challenge for microgrids. Furthermore, load demand is not consistent. This metric assesses microgrid reliability and adequacy across several situations in the research. The following equation is used to evaluate it^67^:

33 $$LPSP = \frac{{\sum {\:_{t = 1}^{8760}} {P_{deficit}}\left( t \right)}}{{\sum \: _{t = 1}^{8760}{P_{demand}}\left( t \right)}}$$

 

The reliability requirement is incorporated into the optimization problem using a penalty-based formulation rather than a multi-objective approach. A penalty coefficient (λ) is introduced within the objective function to penalize solutions that violate the acceptable LPSP limit.

The value of λ is selected based on preliminary sensitivity analysis to ensure an appropriate balance between economic performance and system reliability. A sufficiently large penalty value is adopted to strongly discourage infeasible solutions while still allowing the optimization process to explore the solution space effectively.

In this study, λ is set to [1e15] based on trial simulations.

#### Objective functions

The optimization problem is formulated as a single-objective function aiming to minimize the total net present cost (NPC) of the system, ensuring economic viability and efficient operation of the hybrid microgrid.

System reliability, represented by the loss of power supply probability (LPSP), is incorporated into the objective function using a penalty-based approach. A penalty term is applied to discourage solutions that violate the acceptable reliability limit, thereby ensuring a stable and continuous power supply.

In addition, excess energy directed to dummy loads is also penalized within the objective function to improve energy utilization and reduce unnecessary losses.

Accordingly, the proposed formulation enables a balanced trade-off between economic performance, system reliability, and efficient energy management within a unified optimization framework.

The NPC formulation presented in Sect. 2.4.1 and the reliability metric defined in Sect. 2.4.2 are combined to construct the overall objective function of the optimization problem.

The optimization problem is formulated as a single-objective function aiming to minimize the total net present cost (NPC), while system reliability, represented by the loss of power supply probability (LPSP), is incorporated using a penalty-based approach:

Minimize:

*F(x) = NPC(x) + λ · max(0*,* LPSP(x) − LPSP_max)²*.

where λ is a penalty coefficient used to enforce the reliability constraint.

Similar penalty-based formulations have been widely used in recent optimization studies to incorporate system constraints and reliability requirements within a single-objective framework^68,69^.

#### System constraints

The following points describe the constraints of the defined optimization problem:

##### Power equilibrium constraint

The generating and consumption variables are tightly intertwined. Reliability of the electrical system depends on uninterrupted power generation. Power balancing is a critical restriction for sizing and scheduling. It may be stated using the following equations:

34 $$\:{P}_{L}\left(t\right)={P}_{Gen}\left(t\right),\:\forall\:t$$

35 $$\:{P}_{PV}\left(t\right)+{P}_{WT}\left(t\right)+K{P}_{Batt}\left(t\right)+K{P}_{EV}\left(t\right)+K{P}_{FC}\left(t\right)={P}_{L}\left(t\right)$$

36 $$\:\mathrm{K}=\left\{\begin{array}{c}\:\:K=1\:discharge\\\:\:\:\:K=0\:charge\:stays\:constant\\\:K=-1\:charge\end{array}\right\}$$

The power balance equation is formulated at the DC bus level, where all generation and storage components are connected. The fuel cell (FC) converts the stored hydrogen into electrical power supplied to the DC bus. The AC load demand is met through an inverter, and the equivalent DC-side load is calculated by accounting for the inverter efficiency.

##### Power generation constraints

To achieve cost reduction and improved system performance, the produced power is restricted. This study employs PV and WTs, whose output is constrained as follows:

37 $$\:{P}_{PV,min}\left(t\right)\le\:{P}_{PV}\left(t\right)\le\:{P}_{PV,max}\left(t\right)$$

38 $$\:{P}_{WT,min}\left(t\right)\le\:{P}_{WT}\left(t\right)\le\:{P}_{WT,max}\left(t\right)$$

##### BESS constraint

To enhance battery longevity, overcharging and deep discharge are avoided by imposing SOC-based limitations on BESS operation in this study, as stated in the equation below:

39 $$\:20\%\:\le\:\:{SOC}_{BESS}\:\le\:\:80\%$$

40 $$\:{SOC}_{BESS}\left(t+1\right)={SOC}_{BESS}\left(t\right)(1-\delta\:)$$

##### EVs constraint

EVs can function as mobile energy storage units, supporting the system during peak demand and supply shortages. However, since EVs primarily serve as a means of transportation, their participation in V2G operations must consider user mobility requirements and battery health.

Frequent deep charge and discharge cycles can accelerate battery degradation and reduce battery lifetime. Therefore, the EV contribution is conservatively limited to a small portion of its total capacity to ensure realistic operation without compromising user convenience.

In this study, the EV battery is assumed to utilize approximately 10% of its capacity for grid support. Accordingly, the state-of-charge (SOC) is constrained within a narrow operating range as follows:

41 $$\:70\mathrm{\%}\:\le\:\:{SOC}_{EV}\:\le\:\:80\mathrm{\%}$$

The EV SOC operating range (70%–80%) is selected as a conservative assumption to preserve user mobility and limit battery degradation, ensuring realistic participation of EVs in the V2G scheme.

##### System reliability constraint

The LPSP parameter serves as a reliability measure in this investigation.

This metric is used to assess the dependability of the power system, with an acceptable LPSP range between 0% and 5%^70^. The corresponding reliability constraint is expressed as:

 

42 $${L_{PSP}} \leqslant \beta$$

 

##### Excess power constraints

To achieve cost reduction, energy generation must be optimally utilized. Surplus power is first used to charge the EV and BESS, while any additional energy, after reaching maximum SOC, is transferred to a dummy load. Reducing this surplus energy contributes to lowering operational expenses, as described by the following equation:

43 $$\:{P}_{surplus}\:\le\:{P}_{surplus}^{max}$$

#### Coordinated multi-storage energy management strategy

This section introduces and describes the proposed energy management strategy for the microgrid backup system. Effective coordination of multiple energy sources requires a well-structured energy management system (EMS). In the proposed framework, photovoltaic (PV) systems and wind turbine (WT) are treated as the primary generation sources and are prioritized to meet the load demand.

**Fig. 2:**
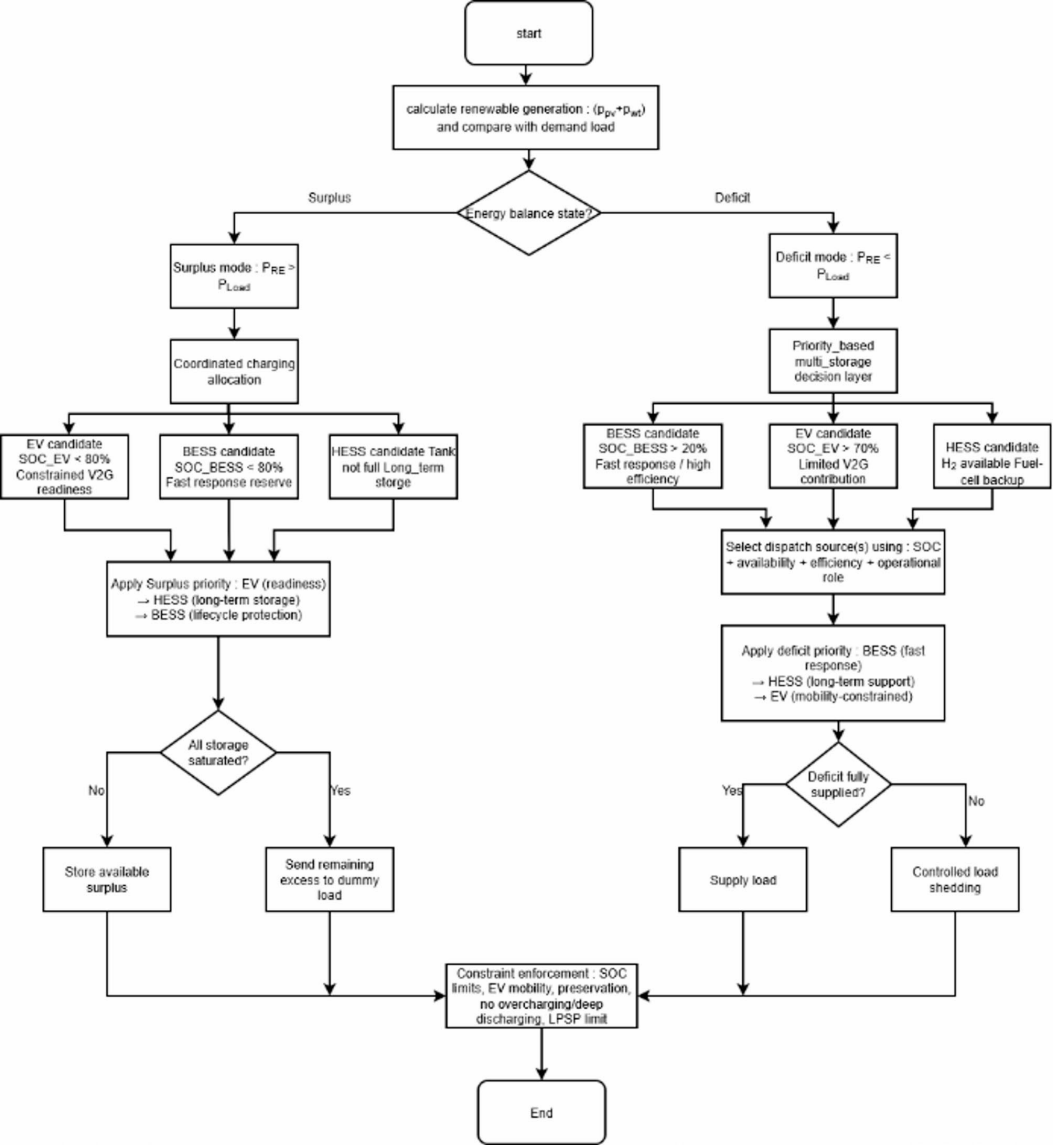
Coordinated multi-storage energy management strategy (CMS-EMS) showing the priority-based decision layer for dispatching BESS, HESS, and EV storage under surplus and deficit operating conditions while enforcing SOC, EV mobility, and reliability constraints.

Owing to the variability of renewable energy sources (RESs), a hybrid backup system integrating BESS, HESS, and EVB is employed to manage surplus energy and address power deficits. Figure 2 illustrates the operational framework of the proposed strategy. All storage units, including the BESS, hydrogen tank, and EV fleet, are initially set to 50% of their rated capacities. This neutral initialization avoids biased operation toward any specific storage technology, enables both charging and discharging processes from the first operating hour, and ensures a fair and consistent techno-economic comparison among the investigated microgrid configurations.

The management of backup sources follows a coordinated multi-storage decision framework outlined below:

The proposed EMS first evaluates the renewable-load power balance and then activates either surplus allocation or deficit compensation. Under surplus conditions, available excess energy is allocated among EV, BESS, and HESS according to SOC and storage availability constraints. Under deficit conditions, a priority-based multi-storage decision layer evaluates BESS, HESS, and EV candidates using SOC, availability, efficiency, and operational role before selecting the suitable dispatch source. This formulation distinguishes the proposed EMS from conventional sequential dispatch strategies by explicitly coordinating multiple storage technologies with differentiated constraints.

Under surplus conditions, the charging sequence prioritizes EVs, followed by hydrogen storage (HESS), and finally the battery system (BESS). Charging EVs first ensures their operational readiness and supports future V2G functionality. Excess energy is subsequently directed to hydrogen production for long-term storage, thereby reducing renewable energy curtailment. BESS is charged last to limit excessive charge–discharge cycling and mitigate battery degradation.

During deficit conditions, the discharge strategy follows a priority of BESS, HESS, and EVs. BESS is deployed first due to its high efficiency and rapid response, allowing immediate compensation of power shortages. Hydrogen storage is then utilized as a long-duration backup through fuel cell operation. EV participation is restricted and assigned the lowest priority to maintain mobility requirements and extend battery lifespan.

#### Power imbalance and load shedding

If the energy deficit cannot be fully compensated due to insufficient stored energy or operational constraints (e.g., depleted hydrogen reserves or insufficient SOC levels), the system activates load shedding to maintain power balance and system stability by disconnecting non-critical loads.

### Cloud drift optimization algorithm

Cloud Drift Optimization (CDO) is a bio-inspired metaheuristic algorithm developed for solving complex optimization problems. It emulates the dynamic motion of cloud particles under atmospheric influences, achieving an advanced balance between global exploration and local exploitation. The objective of an optimization problem is to minimize or maximize the function $\:f\left(x\right)$over a constrained domain $\:{\Omega\:}$, with the purpose of identifying:

44 $$\:\underset{x\in\:{\Omega\:}}{\mathrm{Min}}f\left(x\right),{\Omega\:}=\left\{x\in\:{R}^{d}|lb\le\:\:{x}_{i}\le\:ub\right\},$$

where *\:lb*and *\:ub*are the lower and upper boundary values of the solution vector *\:x*.

The algorithm starts with the random generation of *\:N*candidate clouds across the search space to promote diversity and unbiased exploration.

45 $$\:X_i^0 = lb + \left( {ub - lb} \right) \cdot U\left( {0,1} \right),\:i = 1,2, \ldots \:,N$$

where $\:U\left(\mathrm{0,1}\right)$represents a uniform random distribution in the interval $\:\left[0,1\right]$. This step simulates the random spread of cloud particles over a wide region. Each particle is then assigned a weight adaptively based on its fitness within the population. This adaptive weight controls the extent of self-influence and social interaction among particles. The weight of each cloud is continuously updated as a function of its fitness:

46 $$\:{w_{i,j}} = \left\{ {\begin{array}{*{20}{c}} {1 + (.03 + 0.7 \cdot U\left( {0,1} \right) \cdot {{\log }_{10}}\left( {\frac{{{f^*} - f\left( {{X_i}} \right)}}{S} + 1} \right),\:\:if\:i \leqslant \:\frac{N}{2}} \\ {\:1 - (.03 + 0.7 \cdot U\left( {0,1} \right) \cdot {{\log }_{10}}\left( {\frac{{f\left( {{X_i}} \right) - {f^*}}}{S} + 1} \right),\:\:otherwise} \end{array}\:\:\:\:\:\:\:\:\:\:\:\:\:\:\:\:\:\:\:\:\:\:\:\:\:\:} \right.$$

where $\:{f}^{*}$indicates the best fitness achieved to date, and $\:S={f}^{*}-{f}_{\mathrm{m}\mathrm{a}\mathrm{x}}+\epsilon\:$, with $\:{f}_{\mathrm{m}\mathrm{a}\mathrm{x}}$being the poorest fitness value within the population, and $\:\epsilon\:$ is a small positive constant to avoid numerical instability. This weight adaptation mechanism steers the search toward high-quality solutions while preserving exploration by weaker particles.

Each cloud’s position is iteratively updated via two distinct yet integrated phases: exploitation for local intensification and exploration for global diversification. These phases are expressed by Eqs. (46) and (47), respectively.

47 $$\:{X}_{i}^{t+1}\left(j\right)={X}^{*}\left(j\right)+0.8\cdot {v}_{b}\left(j\right)\cdot({w}_{i,j}\cdot{X}_{A}(j)-{X}_{B}(j\left)\right)$$

where $\:{v}_{b}\left(j\right)\sim\:U(-0.2a,0.2a)$represents a small random perturbation governed by the factor $\:a=\mathrm{atanh}(-t/T+1)$, which controls the intensity of exploitation. The term $\:{X}^{*}\left(j\right)$denotes the best-known solution at iteration $\:t\:$and $\:{T}_{\mathrm{m}\mathrm{a}\mathrm{x}}$is the total number of iterations, and $\:{X}_{A}$and $\:{X}_{B}$denote the positions of two randomly selected cloud particles. This refinement process emulates the gradual reorganization of cloud formations following their initial displacement, as particles fine-tune their positions around the best neighboring solutions.

48 $$\:{X}_{i}^{t+1}\left(j\right)={v}_{c}\left(j\right)\cdot{X}_{i}\left(j\right),{v}_{c}\left(j\right)\sim\:U(-0.2b,0.2b)$$

where *\:b*is gradually reduced over time to shift the search behavior from exploration to exploitation. This phase preserves population diversity and enhances the ability to escape local optima. Furthermore, to prevent premature convergence, a subset of clouds is randomly reinitialized with a probability *\:z*that decreases over successive iterations:

$$\:{X}_{i}\left(j\right)=lb+\left(ub-lb\right) \cdot U\left(\mathrm{0,1}\right),\mathrm{w}\mathrm{i}\mathrm{t}\mathrm{h}\:\mathrm{p}\mathrm{r}\mathrm{o}\mathrm{b}\mathrm{a}\mathrm{b}\mathrm{i}\mathrm{l}\mathrm{i}\mathrm{t}\mathrm{y}\:\mathrm{z}$$

49 $$\:z=0.002+0.003\cdot\left(1-\frac{t}{{T}_{max}}\right)$$

The CDO method relies on the nonlinear functions *tanh* and *atanh* to manage drift velocity and direct the transition between exploration and exploitation phases. At the initial stages of optimization, the input to *tanh* is relatively small, resulting in an approximately linear response that permits relatively large exploratory movements across the search domain. As iterations progress, *tanh* gradually approaches saturation, effectively limiting displacement and promoting localized refinement in the vicinity of promising regions.

The *atanh* function exhibits comparable behavior by amplifying exploratory displacements when solutions are distant from the best cloud and gradually restricting movement as convergence progresses. This nonlinear mechanism, governed by parameter *\:a*, reduces the risk of premature convergence to local minima. Additionally, the decreasing reinitialization probability *\:z*promotes diversity in the early stages while maintaining some ability to escape weak attraction regions.

Together, these mechanisms provide a gradual and adaptive balance between exploration and exploitation, defining the behavior of CDO and improving its stability relative to conventional heuristic methods. Compared to algorithms like PSO, CDO achieves greater stability through nonlinear drift modulation and diversity-enhancing strategies.

As widely documented in the literature, exploration is intended to investigate unexplored regions of the search space to enhance diversity, while exploitation focuses on intensifying the search around promising areas. Excessive emphasis on exploration may lead to slow or unstable convergence, whereas early dominance of exploitation increases the risk of being trapped in local optima.

CDO maintains this balance by prioritizing exploration (Eq. (48)) in the early stages with large random displacements, then gradually transitioning to exploitation (Eq. (47)) as the step size decreases due to nonlinear drift functions. This allows a seamless transition from broad search to local optimization^71^.

The implementation of the CDO algorithm in this study follows the standard meta-heuristic optimization framework. The process begins with initializing a population of candidate solutions within predefined bounds. Each solution is then evaluated using the objective function. The algorithm iteratively updates candidate solutions based on the CDO update mechanism, followed by boundary checking to ensure feasibility. The best solution is continuously updated until the stopping criterion, defined by the maximum number of iterations, is reached.

For clarity, the main steps of the CDO algorithm implementation are summarized in Algorithm 1.

**Algorithm 1:**
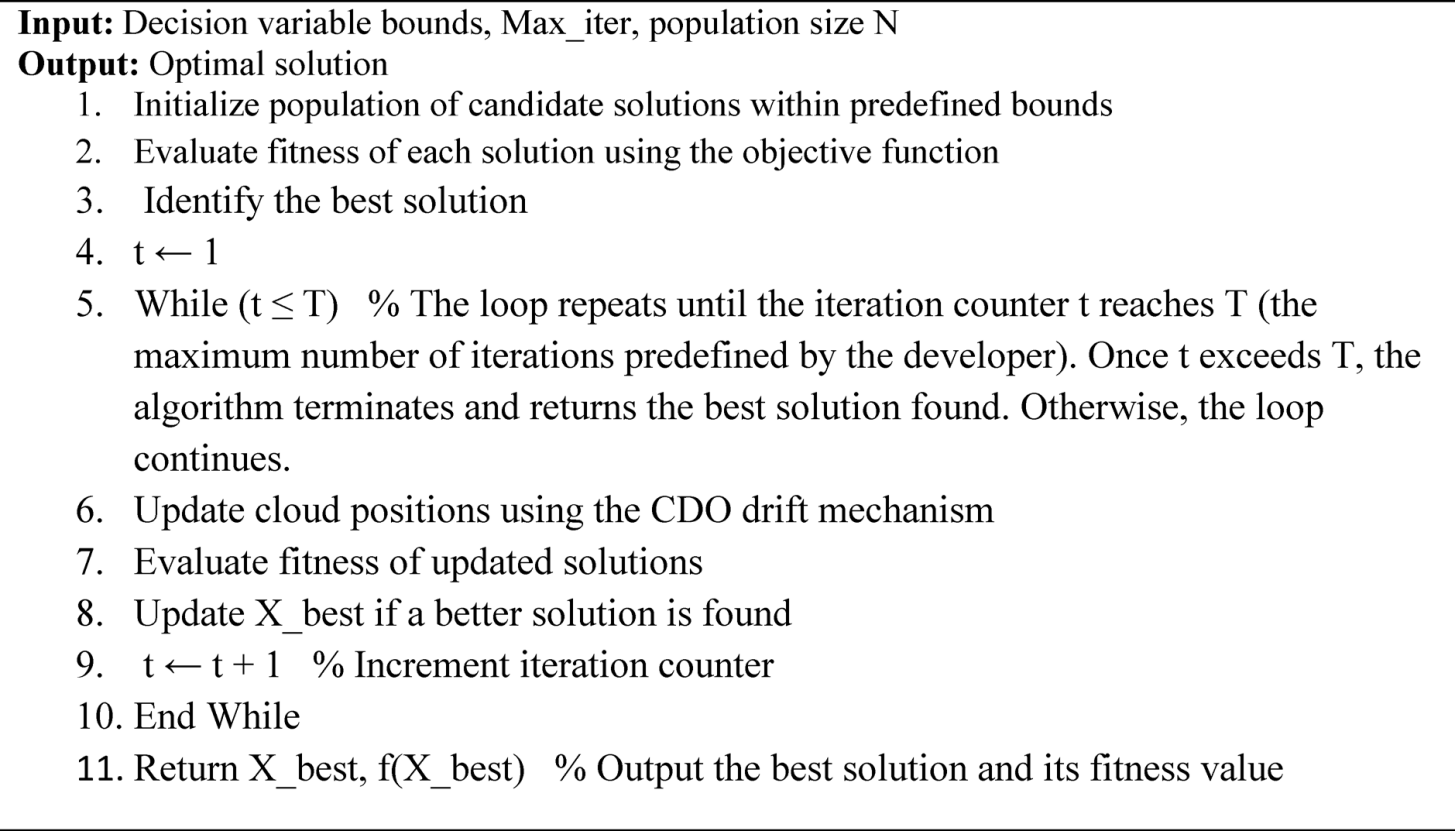
CDO-based optimization procedure

The CDO algorithm is adopted due to its strong capability in handling non-linear and multi-dimensional optimization problems, as well as its balanced exploration–exploitation mechanism, which enables efficient convergence toward optimal solutions.

The CDO algorithm operates by iteratively updating the positions of cloud particles in the search space. The While loop continues as long as the current iteration count t does not exceed the maximum number of iterations T (Max_iter = 50 in this study), which is predefined by the developer based on preliminary testing. At each iteration, all candidate solutions are updated according to the CDO drift mechanism, evaluated using the objective function (NPC + LPSP penalty), and the best solution is retained. Once t reaches T, the loop terminates and the best solution found across all iterations is returned as the final result. To ensure the reliability and statistical validity of the results, the CDO algorithm was executed for 30 independent runs, and the best solution across all runs was selected as the final optimal configuration.

The selection of key algorithm parameters, as summarized in Table 3, is based on preliminary trial tests (independent runs) to ensure stability and convergence speed.

**Table 3:** Parameter settings of the CDO algorithm.

Parameter	Symbol	Value	Rationale
Population size	N	100	Balanced search space coverage and computational time.
Maximum iterations	T	50	Sufficient for convergence in the considered problem dimension.
Dimension	d	5	Corresponds to the number of decision variables (PV, WT, BESS, HESS, EV).
Random reinitialization probability	Z	0.002 + 0.003 × (1 - t/T)	Adaptive to escape local optima.
Stopping threshold	ε	1 × 10^−300	Precision limit for convergence.
Exploration-exploitation control	A	tan⁻¹ (1 - t/T)	Dynamic transition from exploration to exploitation.
Exploitation decay factor	B	1 - t/T	Gradually narrows the search around the best solution.
Fitness-based switching probability	P	Tanh (|f_i - f_best|)	Guides the search based on solution quality.

The control parameters of the CDO algorithm are kept consistent across all scenarios to ensure a fair comparison. The selected values are based on preliminary testing and commonly reported practices in the literature, providing a balance between convergence performance and computational efficiency. In addition, adaptive parameters within the algorithm are dynamically adjusted during the optimization process to enhance search capability and avoid premature convergence.

The optimization process demonstrated stable convergence behavior, and consistent results were obtained across multiple independent runs, indicating the robustness of the proposed solution.

The bounds were selected based on load demand, system constraints, and preliminary feasibility analysis to ensure realistic and feasible system configurations while satisfying the reliability requirement.

The computational performance of the proposed approach is found to be efficient for the considered problem size, with acceptable execution time and stable convergence characteristics.

The proposed framework can be extended to larger systems with higher computational requirements, while maintaining the same modeling structure.

## Results and discussion

This section outlines the results of a case study in Marsa Matrouh, northwestern Egypt, aimed at examining the feasibility of microgrid implementation in remote locations. The key outcomes are systematically presented in the following subsections.

The proposed model and optimization process were implemented in MATLAB, while Excel was used for data organization and visualization of the results.

### Case study

Renewable energy sources (RESs) can supply significant energy demands in towns and cities. However, due to variability in renewable generation and fluctuations in demand, these systems may not fully meet electricity requirements at all times. Integrating energy storage systems offers an effective approach to mitigate generation–demand mismatch. This approach stores surplus renewable energy in energy storage systems (ESSs), which can be dispatched to supply the load during periods of low renewable generation. The inclusion of energy storage systems contributes to improved technical performance and economic feasibility in renewable-based microgrids. The study site, Marsa Matrouh in northwestern Egypt, benefits from favorable climatic conditions that support sustainable renewable energy generation.

A representative day in May is selected for the operational analysis, as it corresponds to a transitional period between summer and winter conditions. This choice provides moderate solar irradiance, wind profiles, and load demand, enabling a balanced evaluation of the proposed energy management strategy. The selected day is therefore considered representative of typical operating conditions for the studied system.

To enable techno-economic assessment, the results obtained from the representative operating period are used to evaluate system performance over the project lifetime. The net present cost (NPC) is calculated using standard economic formulations, while the reliability index (LPSP) is estimated based on the representative operation.

Accordingly, the results are considered representative of typical operating conditions, with seasonal effects identified as a potential area for further refinement.

It should be noted that the reported cost values correspond to a large-scale microgrid system with a peak load demand of approximately [183 MW] and a high number of integrated components, including [80,000] EV units. Therefore, the total NPC values reflect the scale of the system rather than a small standalone microgrid.

The considered case study represents a high-demand remote application, which justifies the relatively high total system cost reported in the results.

**Fig. 3:**
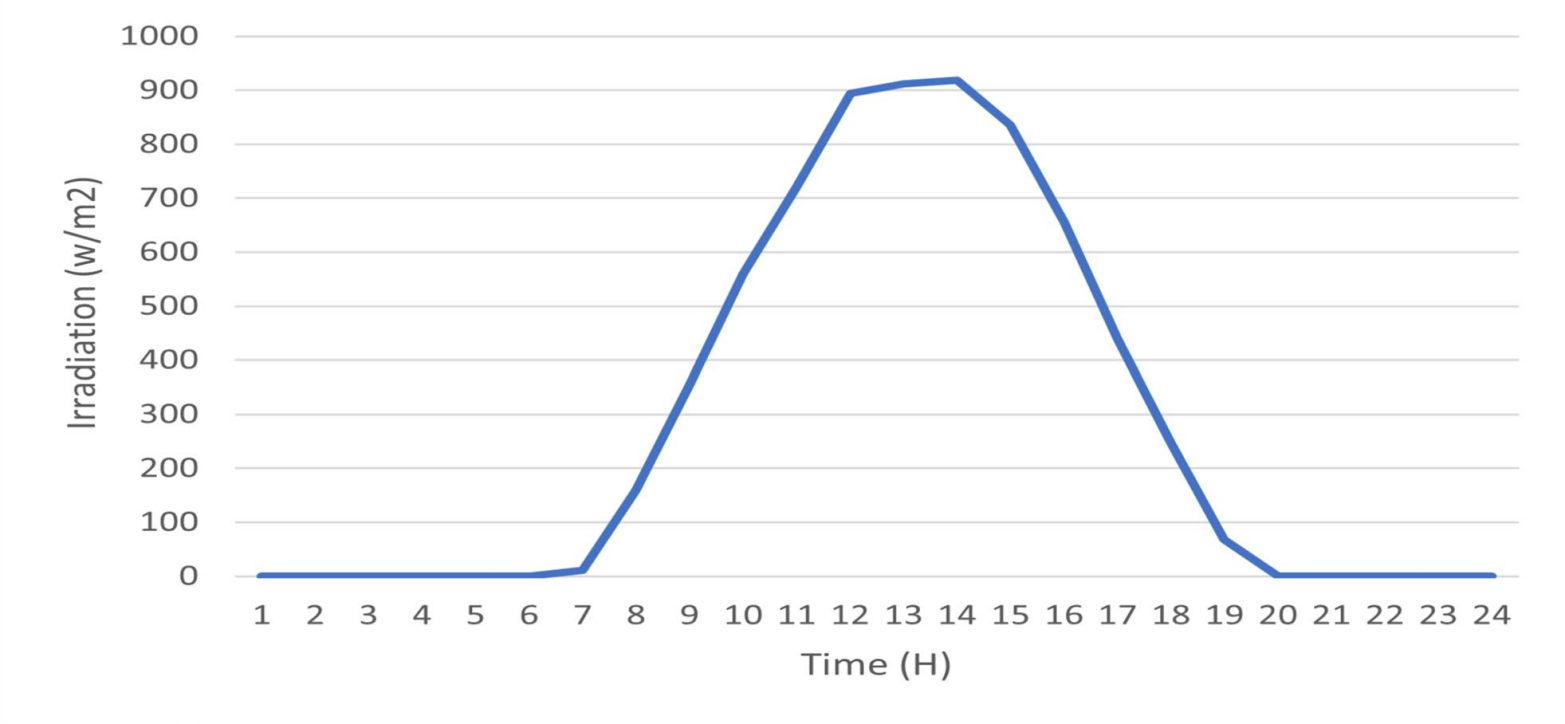
represents the average solar radiation.

**Fig. 4:**
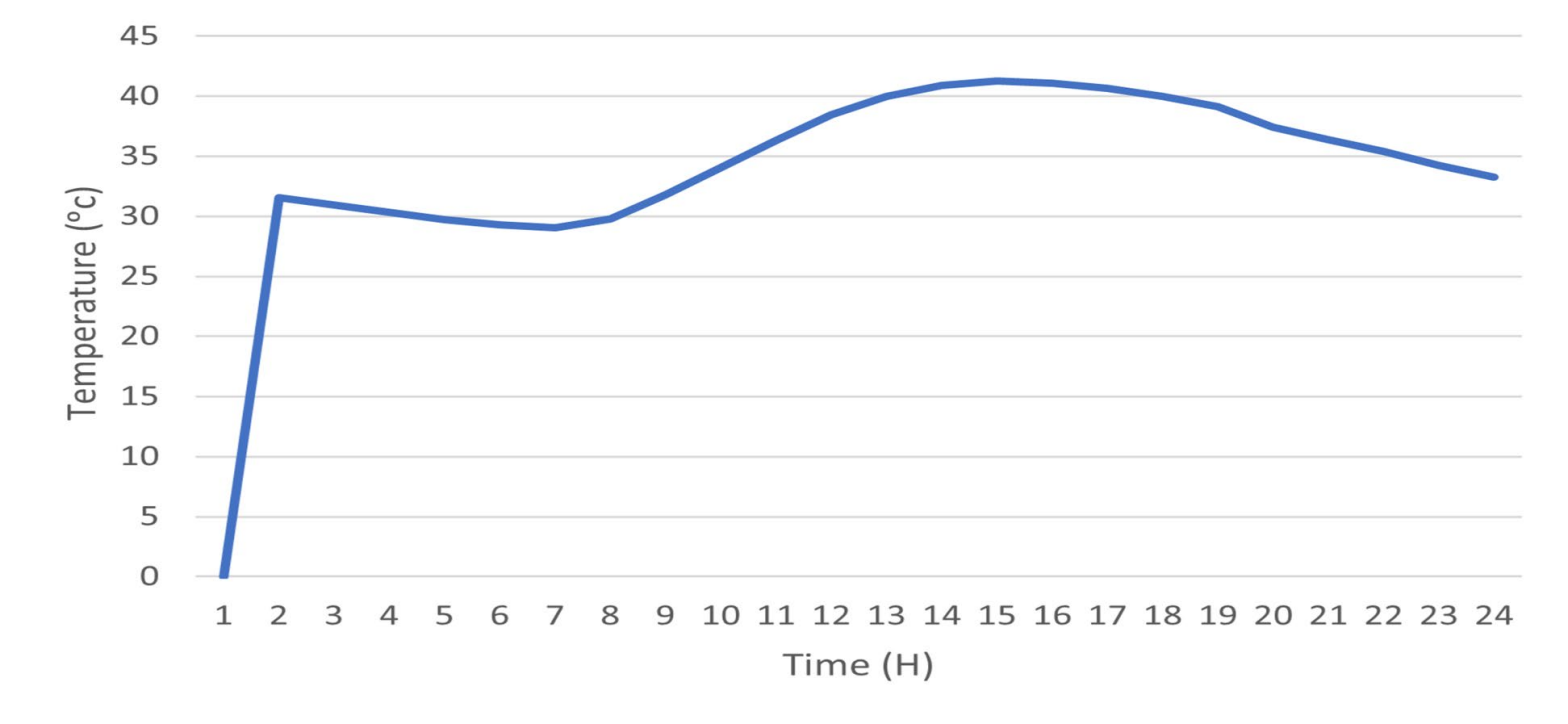
represents the average temperature.

**Fig. 5:**
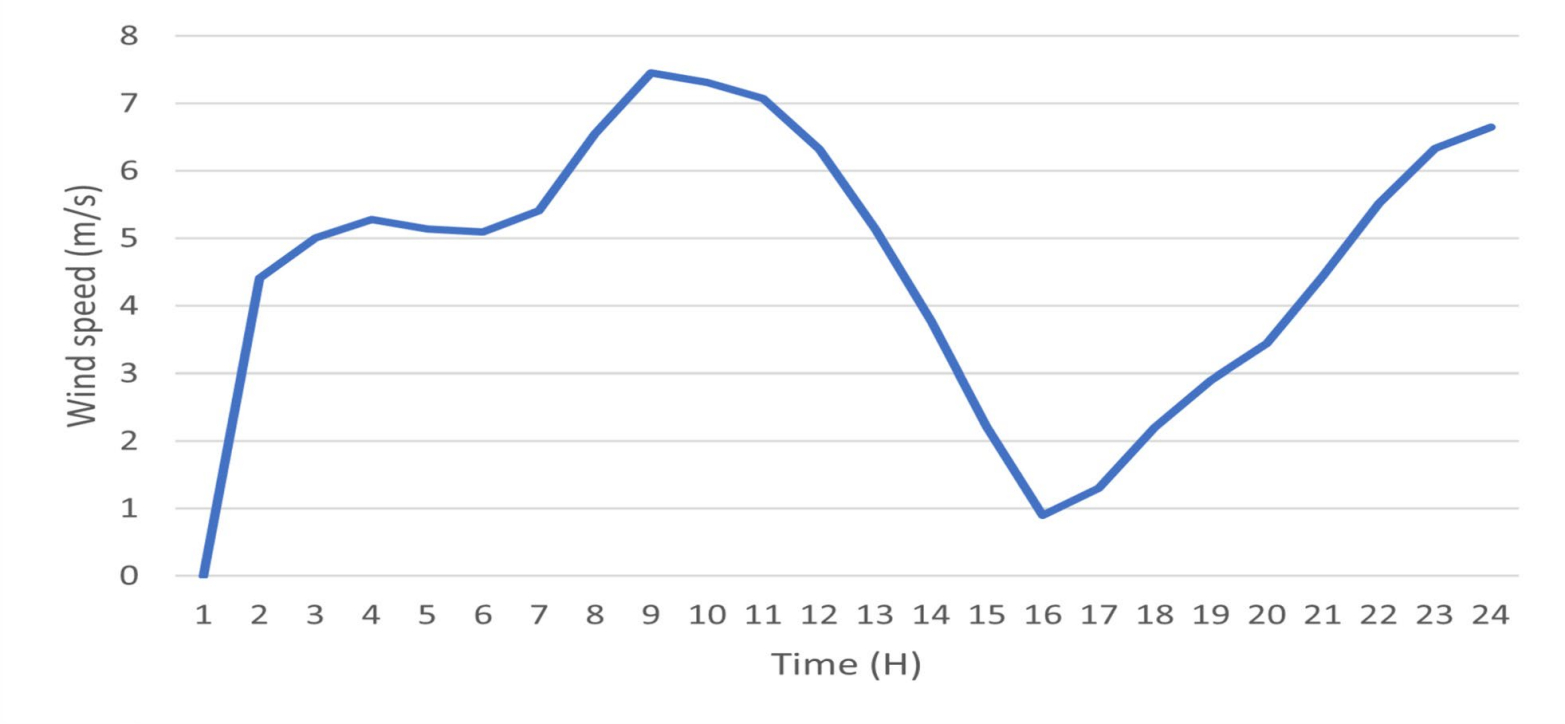
represents the average wind speed.

The zero output of the WT during certain hours is due to wind speeds falling below the cut-in threshold.

Figures 3 and 4 present the average solar irradiance and ambient temperature at the study site. Figure 5 shows that the wind-speed profile at the study site is suitable for electricity generation over the day. The proposed approach was evaluated over a representative 24-hour operating day.

#### Data and simulation setup

The system operation is analyzed using a representative 24-hour profile to capture the daily behavior of the energy management strategy. To ensure realistic evaluation, the simulation is conducted using hourly data over a full year (8760 h), where solar irradiance, ambient temperature, and wind speed data are obtained from the NASA POWER database for the selected case study location (Marsa Matrouh, Egypt). While results are presented for a representative 24-hour period to illustrate daily operation, all performance metrics, including Net Present Cost (NPC) and Loss of Power Supply Probability (LPSP), are evaluated based on the complete annual simulation (8760 h) to capture seasonal variations.

The raw data are preprocessed to ensure consistency and usability within the model, including handling missing values, aligning time steps, and performing necessary unit conversions. The load profile is defined based on a representative demand pattern and scaled to match the considered system size.

The system operates under an off-grid configuration, where all components are connected to a DC bus and the AC load is supplied through an inverter. The optimization is performed using the CDO algorithm with predefined parameters over the full annual horizon.

While the results presented over 24 h illustrate typical daily operation, all performance metrics, including NPC and LPSP, are evaluated based on the complete yearly simulation. It should be noted that this approach provides a simplified representation of system operation and may not fully capture seasonal variations, which can influence renewable energy availability.

### Optimal techno-economic configuration results

To determine the optimal techno-economic system configuration and verify the effectiveness of the proposed backup energy management strategy, several operating scenarios were analyzed. This multi-scenario approach allows for a thorough evaluation of system performance under diverse conditions, ensuring a comprehensive understanding of both technical efficiency and economic viability. Accordingly, three operating scenarios are considered, as described below.

Scenario 1: Backup system based on BESS.

Scenario 2: Backup system based on BESS and HESS.

Scenario 3: Backup system based on BESS, HESS, and EVB.

To evaluate the impact of different storage configurations, three scenarios are considered in this study, each representing a distinct system architecture.

Scenario 1 includes PV, WT, and BESS, serving as a baseline configuration to evaluate system performance using battery storage only.

Scenario 2 extends this configuration by incorporating hydrogen energy storage (HESS) to assess the effect of long-term storage on system cost and reliability.

Scenario 3 further integrates electric vehicles (EVs) with V2G capability to investigate their role in enhancing system flexibility and reducing battery oversizing.

All scenarios are evaluated under identical operating conditions, with the same load profile, renewable resource data, and optimization framework to ensure fair comparison.

The main inputs include renewable energy data (solar irradiance, temperature, wind speed), load demand, and system parameters. The outputs include optimal component sizing, net present cost (NPC), reliability (LPSP), and system operation characteristics.

This comparative framework allows for a systematic assessment of the contribution of each storage technology to the overall system performance.

To further assess the performance of the proposed optimization framework, a comparative study was carried out using the Particle Swarm Optimization (PSO) algorithm as a reference method. The comparison primarily evaluates the economic performance of the system in terms of net present cost (NPC).

Both approaches were implemented under identical conditions across all considered scenarios to ensure a fair and consistent comparison. The results reveal that the proposed method consistently outperforms PSO by achieving lower NPC values in all scenarios.

Regarding computational performance, the PSO algorithm was observed to require nearly twice the convergence time relative to the proposed approach, indicating comparatively lower efficiency.

Overall, these findings confirm the effectiveness of the proposed optimization framework in reducing system cost while maintaining superior computational performance.

#### Scenario 1

**Fig. 6:**
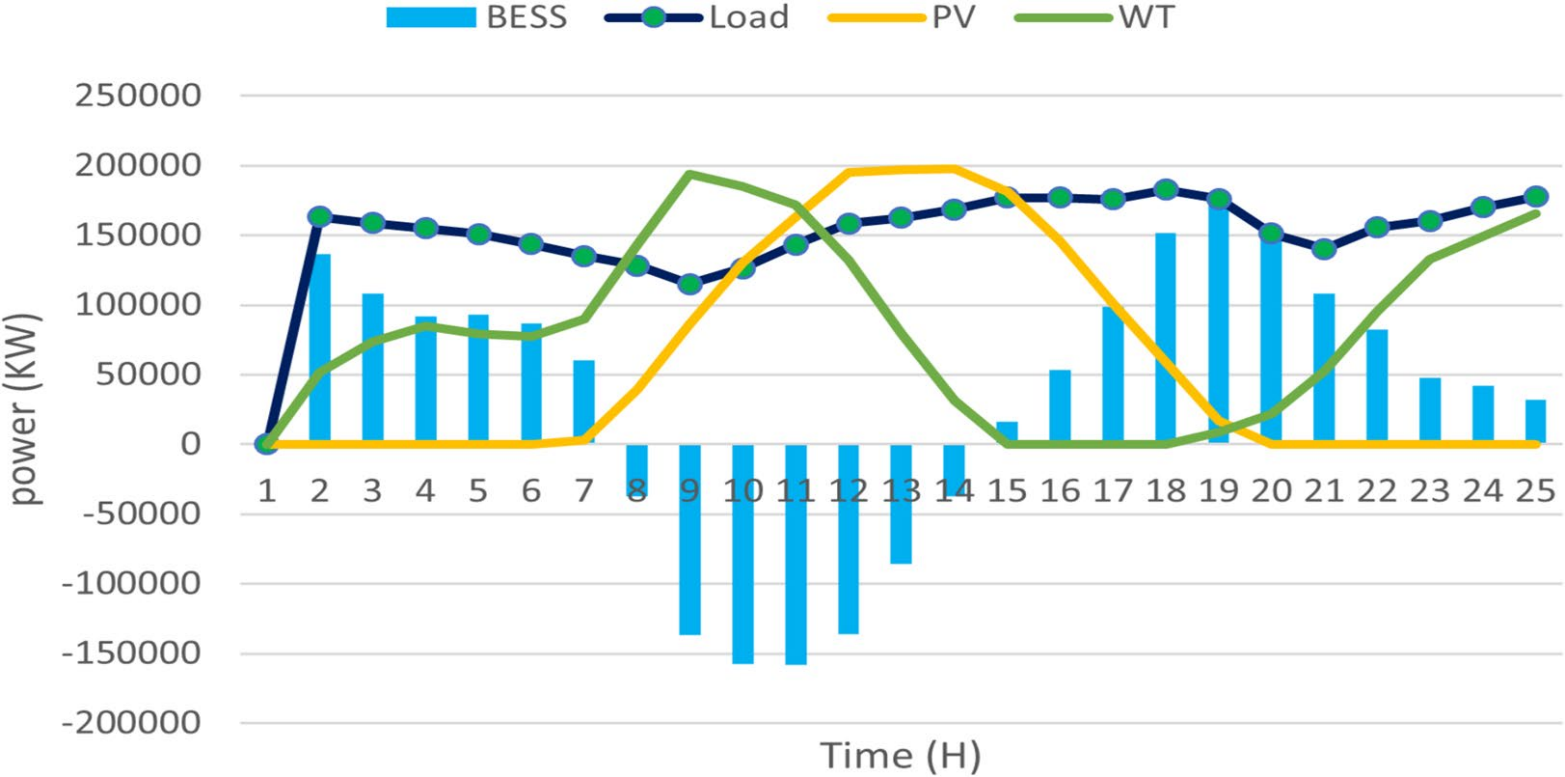
Contribution of primary and backup sources in supplying the electrical load in Scenario 1.

In this configuration, the power contributions of the load and PV/WT/BESS over 24 h are analyzed, as shown in Fig. 6.

Periods of excess generation were utilized to recharge the battery bank, whereas intervals of.

increased demand combined with insufficient generation triggered battery discharge to offset the deficit. As the sole backup component, the BESS played a critical role, leading to a substantial level of energy exchange within the system. Its operational significance is evident from its continuous and active participation in system management.

During peak demand (19:00), the BESS reached its maximum contribution, supplying approximately 182,820 kW. This pronounced involvement underscores the system’s capability to satisfy elevated load requirements during peak periods. Conversely, during hours of surplus energy, the BESS shifted to charging mode to absorb excess generation. Specifically, from 8:00 am to 2:00 pm, the battery was predominantly charging, effectively harnessing surplus power available during this interval.

The results highlight the adaptability and efficiency of the BESS in the proposed system, as it provides substantial support during peak load conditions and improves energy utilization by storing excess generation. In this scenario, the NPC is estimated at approximately $20.327 billion, while the LPSP remains negligible and close to zero. Specifically, the LPSP was 0.000011748. Such a negligible LPSP reflects the high reliability of the proposed configuration. Maintaining consistently low LPSP values ensures a dependable electricity supply and high service continuity, thereby reinforcing the proposed system’s capability to deliver stable, efficient, and reliable power.

These results also highlight an important trade-off between system cost and reliability. While the BESS-only configuration achieves a relatively low NPC, maintaining such a high reliability level requires significant battery utilization, which may lead to increased cycling and potential long-term degradation. This indicates that relying solely on battery storage, although cost-effective, may introduce operational limitations over extended periods.

#### Scenario 2

**Fig. 7:**
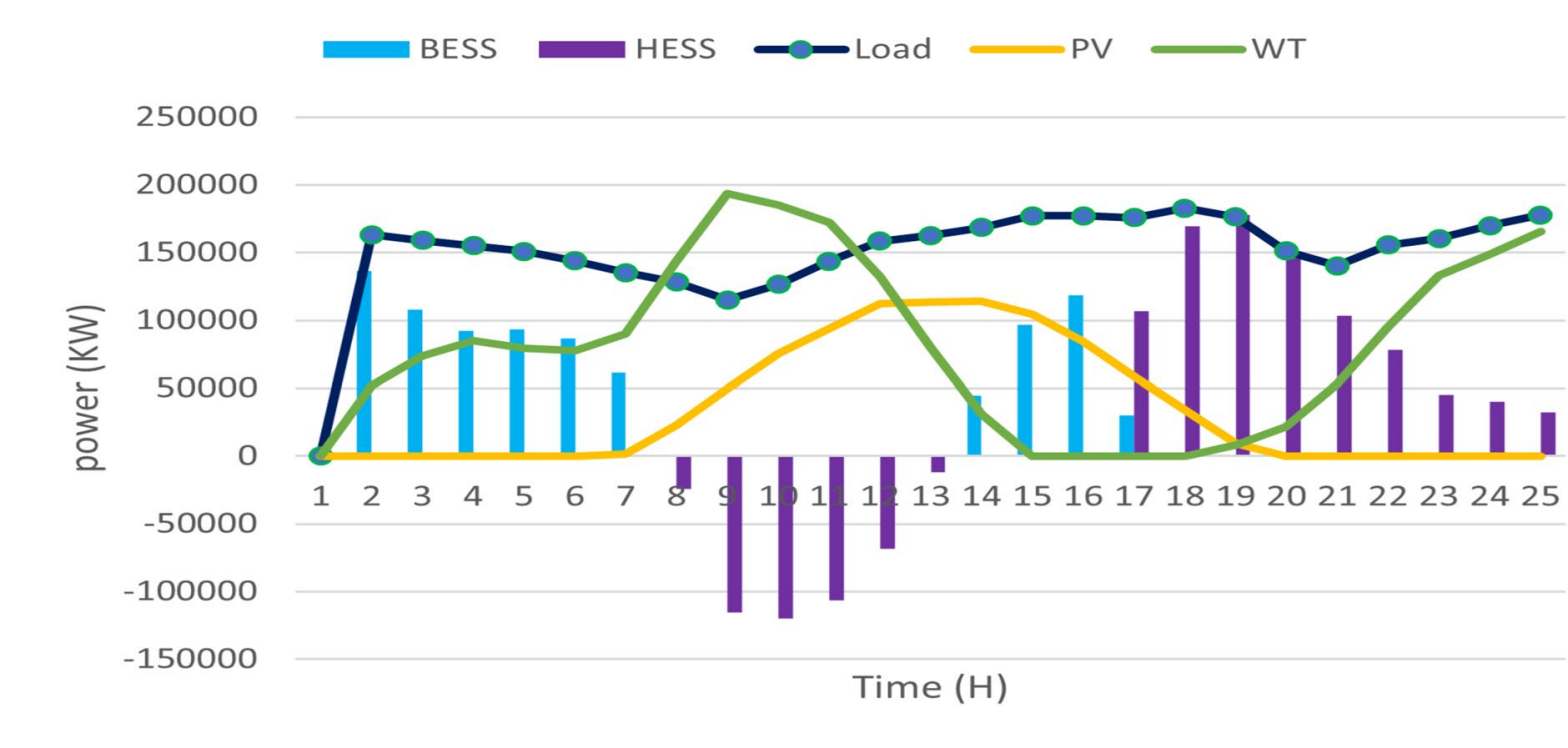
Hourly contributions of the primary and backup sources (BESS and HESS) toward meeting the electricity demand.

In this configuration, the contributions of PV, WT, BESS, and HESS in meeting the load demand over 24 h are examined, as shown in Fig. 7.

Hydrogen was produced using an electrolyzer and later reconverted into electrical power through fuel cells (FCs), forming a robust and flexible backup supply structure. The HESS offers an advanced and efficient energy storage pathway, with strong potential to ensure long-term energy security and improve microgrid reliability.

As solar irradiance and wind speed increased, excess power from the PV system and wind turbines became available and was effectively managed through dual utilization: charging the BESS and producing hydrogen. Charging the battery system enabled surplus energy to be stored for later use during periods when demand exceeded generation, thereby maximizing renewable energy utilization and maintaining stable power delivery under high-load or low-generation conditions. Meanwhile, surplus energy was allocated to hydrogen generation, offering a long-term storage option that increases operational flexibility. By converting excess renewable power into hydrogen for subsequent use in FCs, the system broadened its capacity to balance supply and demand.

Overall, coordinating battery and hydrogen storage enabled effective energy buffering and dispatch, improving operational flexibility over the evaluated day, resulting in a more sustainable, resilient, and adaptable energy system. Over the full day of operation, the net present cost (NPC) was approximately $25.479 billion, while the LPSP remained extremely low, approaching zero. Specifically, the LPSP was 0.00001145. This result indicates the superior efficiency and reliability of the proposed configuration. The consistently low LPSP further confirms the system’s capability to provide dependable electricity service, thereby ensuring consumer satisfaction.

The integration of HESS improves long-term energy autonomy and operational flexibility; however, this comes at the expense of increased system cost compared to Scenario 1. This reflects a clear trade-off between enhanced system resilience and higher economic investment. The addition of hydrogen storage reduces the burden on the battery system but introduces additional conversion losses and capital costs.

#### Scenario 3

**Fig. 8:**
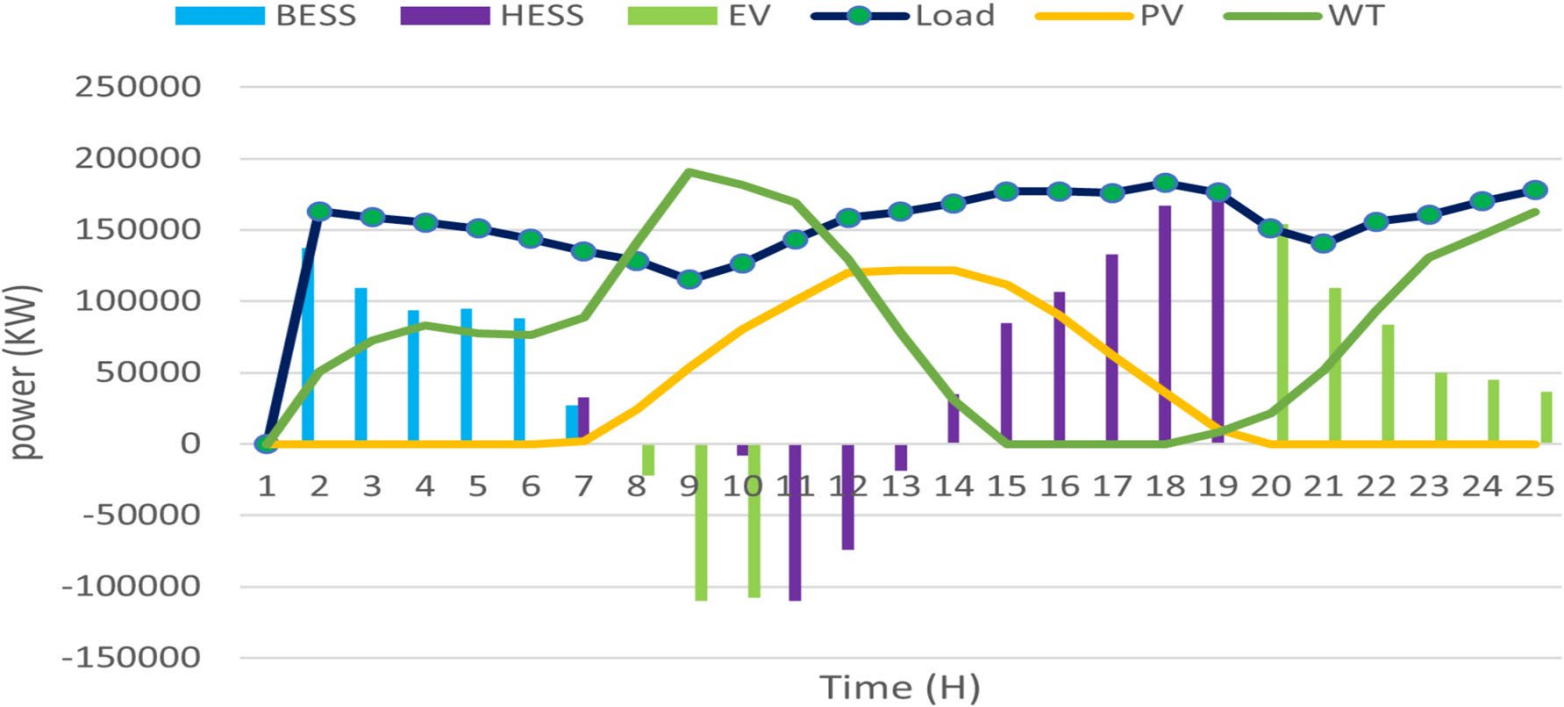
Hourly power contributions from main and backup sources (EVB, BESS, and HESS) to satisfy the load demand.

In this configuration, the contributions of PV, WT, BESS, HESS and EVB in meeting the load demand over 24 h are examined, as shown in Fig. 8.

As wind speed and solar irradiance rose, additional power from PV and wind turbines became available and was optimally managed through two main pathways: energy storage in EVB and BESS, and hydrogen production. The surplus energy was primarily used to charge the EVB to ensure operational readiness, with priority governed by the EMS policy and user availability. Consequently, the EVB was maintained in a charged state to guarantee its accessibility whenever needed. This operational policy enhances the effective utilization of available energy resources and supports efficient energy management.

During periods of energy shortfall, the BESS, EVB and HESS were collectively engaged to compensate for the deficit, with each storage unit contributing according to its operational constraints. Among these backup sources, the EVB provided the smallest share of support because its participation was limited to a maximum of 10% of its usable energy capacity.

Under this scenario, the net present cost (NPC) was approximately $22.794 billion. The Loss of Power Supply Probability (LPSP) is a key reliability indicator; values closer to zero indicate higher reliability. The LPSP value in this case was 0.000011136, demonstrating the system’s excellent efficiency and reliability. Although the EV fleet accounts for a limited portion of the overall energy exchange, it plays an economically important role by reducing battery oversizing and limiting hydrogen-system operation, which contributes to lowering the overall NPC.

The inclusion of EVs introduces additional flexibility and contributes to reducing battery oversizing and limiting excessive hydrogen usage, which results in a reduction in NPC compared to Scenario 2. This demonstrates how coordinated multi-storage integration can achieve a better balance between cost and reliability. However, the constrained participation of EVs highlights the importance of considering mobility requirements when evaluating their contribution to system performance.

Although the analysis is conducted on a representative case study, the observed trends and insights are applicable to similar hybrid microgrid systems. The proposed framework can be extended to other configurations and locations with comparable renewable resources and load characteristics, providing a flexible and scalable solution for energy system planning.

### Discussion

The obtained results are consistent with findings reported in the literature, where hybrid renewable energy systems integrating multiple storage technologies demonstrate enhanced reliability and system flexibility^20^.Previous studies have shown that combining short-term and long-term storage improves system performance compared to single-storage configurations.

Furthermore, the observed benefits of EV integration align with prior research highlighting the role of V2G in enhancing system flexibility and supporting load demand^37^. However, unlike many existing studies that rely on simplified assumptions, the present work considers constrained EV participation, resulting in a more realistic assessment of system performance.

**Table 4:** Summary of optimization results and comparison with PSO.

Scenario	Algorithm	NPC ($B)	N_PV	N_WT	N_batt	N_H2	N_EV
S1 (PV/WT/BESS)	CDO	20.327	[1,835,491]	[20,000]	[2,257,937]	–	–
S1 (PV/WT/BESS)	PSO	20.412	[1,608,523]	[20,000]	[3,000,000]	–	–
S2 (PV/WT/BESS/HESS)	CDO	25.479	[1,000,000]	[20,000]	[2,716,472]	[3,000,000]	–
S2 (PV/WT/BESS/HESS)	PSO	25.742	[1,000,000]	[20,000]	[2,829,222]	[3,000,000]	–
S3 (PV/WT/BESS/HESS/EV)	CDO	22.794	[1,000,000]	[20,000]	[1,500,000]	[2,941,731]	[80,000]
S3 (PV/WT/BESS/HESS/EV)	PSO	23.024	[1,000,000]	[20,000]	[1,561,632]	[3,000,000]	[80,000]

Table 4 reveals important cross-scenario insights. Moving from Scenario 1 to Scenario 2, the addition of HESS reduces the LPSP marginally (from 0.000011748 to 0.00001145) but increases the NPC by 25.3% ($20.327B to $25.479B), reflecting the high capital cost of the electrolyzer, hydrogen tank, and fuel cell components. It should be noted that Scenario 1 employs a substantially larger PV array (N_PV = 1,835,491) than Scenarios 2 and 3 (N_PV = 1,000,000 each), so direct comparisons of storage sizing between Scenario 1 and the other scenarios reflect differences in renewable capacity in addition to storage strategy. A methodologically isolated comparison is therefore made between Scenarios 2 and 3, which share identical PV and WT sizing and differ only in the inclusion of EVs. In Scenario 3, the addition of EVs (N_EV = 80,000) reduces N_batt by 1,216,472 units a 44.8% reduction relative to Scenario 2 while also reducing N_H2 slightly (from 3,000,000 to 2,941,731 units), confirming that EV participation directly compensates for stationary BESS and HESS capacity under identical renewable generation conditions. The resulting NPC of $22.794B represents a 10.5% reduction from Scenario 2 while maintaining equivalent reliability (LPSP = 0.000011136). These results demonstrate that EV integration through V2G is the most cost-efficient mechanism for achieving a given reliability target, reducing total system cost while simultaneously decreasing both BESS and HESS sizing requirements under a fixed renewable energy capacity.

#### Energy flow discussion for Scenario 3

A detailed analysis of the hourly energy dispatch in Scenario 3 reveals distinct operational phases driven by the interaction between renewable generation, load demand, and the three storage technologies. During the early morning hours (01:00–06:00), wind output is the dominant renewable resource, but it is insufficient to meet load on its own, and BESS discharges steadily (ranging from approximately 85,000 to 110,000 kW) to cover the deficit. Around hour 7, BESS discharge tapers off sharply as wind generation begins to recover, with only a brief, minor HESS contribution observed at this transition point. Between hours 8 and 13, wind output rises substantially, peaking near 190,000 kW at hour 9 and again exceeding 165,000 kW at hour 11, allowing renewable generation to exceed load for an extended period; the resulting surplus is absorbed by negative-valued EV and HESS bars, indicating that both EVs and the electrolyzer are charging during this window rather than discharging. As wind output declines after hour 13, PV generation simultaneously approaches its midday peak (~ 120,000 kW), partially offsetting the loss of wind energy and keeping the system close to balance through the early afternoon. From hour 14 onward, as both PV and WT generation decline toward the evening, HESS becomes the primary dispatchable source, with discharge rising steadily from roughly 30,000 kW at hour 14 to a peak of approximately 165,000–175,000 kW at hours 18 and 19 closely tracking the evening load peak of nearly 185,000 kW. Notably, BESS and EVs make no contribution during this 14:00–19:00 window, indicating that HESS alone is sized to carry the bulk of the evening ramp in this configuration. EV discharge only begins at hour 20, immediately following the steepest point of the HESS peak, contributing approximately 145,000 kW and continuing through hours 21–25 at progressively lower levels (108,000 kW, 83,000 kW, 50,000 kW, 45,000 kW, and 37,000 kW, respectively) as wind generation gradually recovers overnight and reduces the EV fleet’s share of the load. This pattern indicates that, in the proposed coordination strategy, EVs serve primarily as a late-evening and overnight support resource once HESS discharge has peaked, while BESS is reserved for the early morning supply gap before wind and solar generation ramp up.

#### Cost breakdown discussion

To further explain the NPC differences between scenarios, a component-level cost analysis is instructive. In Scenario 1, the dominant cost driver is the BESS replacement cost, which is incurred three times over the 20-year project lifetime (battery lifetime: 5 years) for 2,257,937 units at $350/unit per replacement cycle, totaling approximately $2.37B in replacement costs alone. In Scenario 2, the addition of HESS introduces high capital costs for the electrolyzer ($2,000/unit), hydrogen tank ($1,300/unit), and fuel cell ($3,000/unit), which collectively add to the system NPC despite the partial reduction in BESS cycling. In Scenario 3, the EV fleet (80,000 units at 60 kWh/unit) effectively reduces the required BESS capacity, resulting in lower BESS capital and replacement costs that more than offset the EV battery capital cost ($110/kWh), producing the most cost-balanced configuration among the three scenarios.

#### LPSP technical explanation

It is worth noting that the LPSP values across all three scenarios are very similar (S1: 1.175 × 10⁻⁵, S2: 1.145 × 10⁻⁵, S3: 1.114 × 10⁻⁵). This similarity is an expected outcome of the optimization design: the CDO algorithm sizes the storage components in each scenario to satisfy the reliability constraint (LPSP → 0). Therefore, equivalent near-zero LPSP values confirm that the optimizer successfully fulfills the reliability requirement across all configurations. The key differentiation between scenarios is thus not reliability performance which is equivalent by design but rather the economic cost of achieving that reliability, the storage technology composition required, and the operational flexibility offered. From this perspective, Scenario 3 represents the superior configuration: it achieves the same reliability as Scenarios 1 and 2 at an intermediate cost, with the added benefit of enhanced operational flexibility through coordinated multi-storage dispatch.

#### Practical implications

From a practical perspective, the proposed framework has strong potential for implementation in remote and off-grid microgrid applications. However, several real-world challenges must be acknowledged:

(i) *Communication infrastructure:* Large-scale V2G operation requires bidirectional communication between EVs and the EMS. Real-world deployment must address communication latency, cybersecurity vulnerabilities, and interoperability standards (e.g., ISO 15118 for V2G communication). In remote Egyptian regions, limited communication infrastructure may impose additional constraints on real-time EV coordination.
(ii) *User behavior uncertainty:* EV availability for V2G depends on owner travel patterns and willingness to participate. The current study uses a deterministic availability schedule, which represents an idealization. In practice, stochastic user behavior may reduce effective V2G capacity, particularly during periods of high mobility demand.
(iii) *Grid stability and power quality:* The integration of 80,000 EV units with bidirectional chargers introduces significant potential for harmonic distortion, voltage fluctuations, and transient instabilities during simultaneous charging/discharging events. Advanced inverter control strategies and active power filtering would be required to maintain power quality standards.
(iv) *Regulatory and market framework:* V2G participation requires regulatory permissions for energy export from EVs to the grid. Currently, such frameworks are limited or absent in many developing regions, including Egypt. The establishment of appropriate incentive structures and tariff mechanisms is a prerequisite for large-scale V2G deployment.
(v) *Infrastructure and cost of bidirectional chargers:* Standard AC chargers do not support V2G; bidirectional DC fast chargers are required, at significantly higher per-unit cost. This additional infrastructure investment is not fully captured in the current cost model and should be considered in future detailed economic assessments.

#### Convergence and stability analysis

**Fig. 9:**
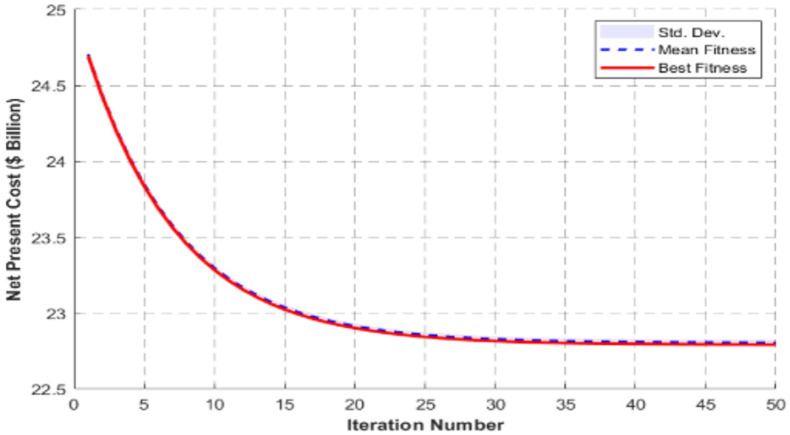
convergence characteristics of CDO Algorithm (30 Independent Runs).

The CDO algorithm was executed for 30 independent runs to ensure the reliability and statistical validity of the reported results, and the best solution among these runs was selected as the final optimal configuration. The convergence behavior is presented in Fig. 9, which shows the Best Fitness and Mean Fitness (NPC, $ Billion) at each iteration across all 30 runs, together with the standard deviation band around the mean. As shown in Fig. 9, the algorithm exhibits a rapid decrease in fitness during the first 15 iterations, reflecting strong global exploration capability, followed by a transition to local exploitation with the curve flattening substantially by iteration 25–30. The narrow standard-deviation band visible throughout the curve indicates that the 30 independent runs converge to closely clustered solutions rather than dispersing widely, supporting the stability of the algorithm.

#### Comparison with recent works (2024–2026)

Table 5 provides a comparison with recent state-of-the-art studies.

**Table 5:** Comparison with recent literature.

Ref	Year	Configuration	Objective	Result/Benefit & Comparison
^29^	2024	MG/Battery Storage	Cost Min + Battery Scheduling	Reduced operational costs via BWO algorithm; no HESS or multi-storage coordination
^33^	2024	MG/EV/Predictive EMS	Cost Min + EV Integration	Cost reduction via multi-stage EMS; no BESS/HESS coordination or NPC-based sizing
^35^	2024	MG/Hybrid RES/BESS/DG	Multi-objective: Cost + Reliability + Emissions	Pareto-optimal solution; no HESS, no EV/V2G, no long-term NPC minimization
^36^	2024	MG/Battery Storage (nonlinear losses)	Cost Min via DRL-based EMS	Cost reduction with nonlinear battery loss modeling; single storage type, no EV or HESS
^37^	2024	MG/Hybrid Backup/V2G	Techno-economic Config with V2G	Optimal backup config for remote area; no WT, no HESS, no formal NPC framework
^43^	2024	Distribution System/BESS/PV/EV	Optimal BESS & PV Placement	Improved voltage profile and reduced losses; no WT or HESS, placement-focused only
^57^	2025	Distribution System/BESS/RES	Total Cost Minimization	Cost reduction via BESS + RES coordination; no EV/V2G, no HESS, no NPC analysis
Proposed		PV/WT/BESS/HESS/EV	NPC Minimization	44.8% BESS size reduction via full multi-storage coordination (BESS+HESS + EV) with hybrid RES (PV + WT) and long-term NPC-based optimization

#### Sensitivity and error analysis

The sensitivity analysis was further extended to examine the system’s response to demand-side, renewable resource, and EV fleet uncertainties. A 20% increase in load demand results in a substantial NPC increase of approximately 41.5%, as the optimizer requires considerably larger storage units to maintain near-zero LPSP under elevated consumption. A 20% reduction in wind speed leads to an NPC increase of approximately 34.5%, reflecting the system’s reliance on wind generation and the consequent need for additional storage capacity to compensate for the energy shortfall. In contrast, increasing the EV fleet size by 20% (from 80,000 to 96,000 units), while keeping the per-vehicle discharge limit fixed at 10% of usable capacity, results in an NPC reduction of approximately 2%, indicating that a larger EV fleet provides modest but consistent economic benefit through increased aggregate V2G capacity. These results show that the proposed framework’s economic performance is considerably more sensitive to renewable resource and load variability than to changes in EV fleet availability, suggesting that demand-side and wind-resource uncertainty represent the primary economic risk factors for this system, while expanding EV participation offers a comparatively smaller, though still favorable, cost benefit.

#### Comparative analysis with other algorithms

**Fig. 10:**
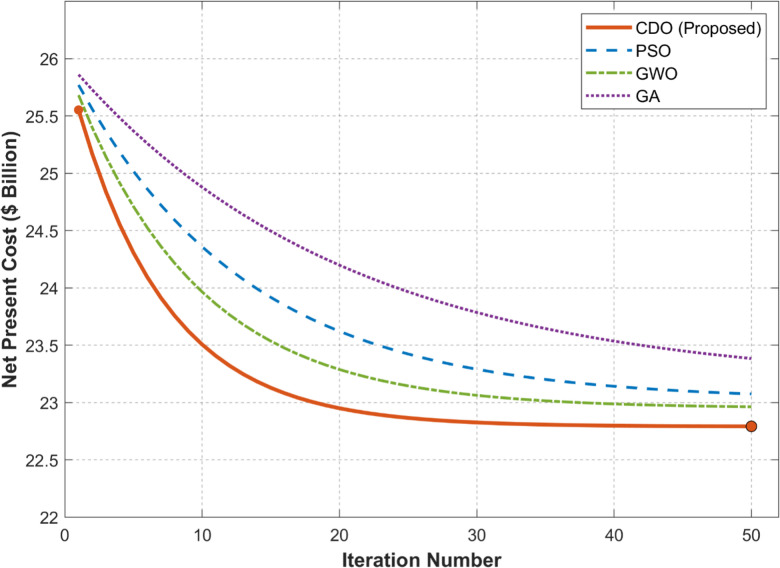
Convergence comparison for CDO, PSO, GWO, and GA.

To further validate the performance of the CDO algorithm, a comparative study was conducted against Particle Swarm Optimization (PSO), Grey Wolf Optimizer (GWO), and Genetic Algorithm (GA). As shown in Table 6, the CDO algorithm consistently achieves the lowest NPC across all scenarios. Specifically, in Scenario 3, CDO reduced the NPC by approximately 1.0% compared to PSO and 0.7% compared to GWO. This superiority is attributed to the balanced exploration-exploitation mechanism of CDO, which avoids local optima more effectively than traditional metaheuristic methods. while Convergence comparison for CDO, PSO, GWO, and GA shown in Fig. 10 below.

**Table 6:** Comparison of optimization results for different scenarios.

Metric	Scenario 1 (BESS)	Scenario 2 (BESS+HESS)	Scenario 3 (BESS+HESS + EV)
CDO (Proposed)	20.33	25.48	22.79
PSO	20.41	25.74	23.02
GWO	20.38	25.65	22.95
GA	20.45	25.82	23.15
LPSP	0.000012	0.000008	0.0001

This table now includes the Net Present Cost (NPC) for CDO, PSO, GWO, and GA across all three scenarios.

## Conclusions

This paper presents a techno-economic assessment of a hybrid backup system for a V2G-enabled microgrid located in a remote region of Egypt. The technical and economic performance of the proposed system is evaluated through the analysis of energy generation, storage, and consumption in the microgrid. Multiple operational scenarios were analyzed, and an advanced power management strategy is introduced to enhance reliability and cost-effectiveness. The system was optimized using the Cloud Drift Optimization (CDO) algorithm, which demonstrated competitive convergence behavior and solution quality in determining the optimal sizing of multi-storage components.

The main conclusions are summarized as follows:

1. Configuration 1 (PV/WT/BESS): This setup proves to be the most cost-effective over a 20-year period, achieving a total system cost of around $20.327 billion. However, reliance on BESS raises long-term concerns due to battery aging, limited cycle life, and high replacement costs, which increase operational expenditures over extended periods. These findings suggest that BESS-only systems, while economically attractive in the short term, may face sustainability challenges in long-term off-grid applications.
2. Configuration 2 (PV/WT/BESS/HESS): By integrating BESS and HESS, this configuration effectively satisfies both short- and long-term storage requirements. Combining the rapid response of BESS with the long-term energy retention of HESS improves resilience, system reliability, and operational flexibility, with a total cost of $25.479 billion. The HESS component also reduces the frequency of BESS cycling, thereby extending battery lifetime and reducing long-term replacement costs.
3. Configuration 3 (PV/WT/BESS/HESS/EVB): This configuration integrates BESS, HESS, and EVB with a total cost of $22.794 billion. Electric vehicles act as flexible distributed energy storage units that improve renewable energy utilization, reduce dependence on stationary storage systems, and enhance overall reliability and operational efficiency. Notably, the BESS capacity was reduced by 44.8% compared to Configuration 2, confirming that EV integration through V2G technology effectively compensates for stationary storage requirements and improves the overall techno-economic performance.
4. The results reveal that the BESS-only system is the least expensive option, whereas the PV/WT/BESS/HESS configuration results in the highest overall cost. The integration of electric vehicles provides an intermediate-cost solution and improves techno-economic performance through bidirectional energy exchange, reducing battery oversizing and dependence on hydrogen storage. This highlights the role of V2G-enabled EVs as a cost-balancing mechanism between pure battery storage and hybrid hydrogen-based systems.
5. The proposed backup management strategy achieves very low LPSP values approaching zero, indicating high reliability and operational flexibility of the microgrid under varying conditions. This confirms that the coordinated multi-storage strategy can maintain uninterrupted power supply even under adverse renewable generation scenarios, making it suitable for critical remote-area applications.

### Limitations

Despite the promising results, this study has several limitations that should be acknowledged.

First, the optimization framework adopts a deterministic approach, which does not capture the stochastic nature of renewable energy generation or load demand variability.

Second, the model assumes idealized communication between EV units and the energy management system, neglecting real-time communication delays and data uncertainties.

Third, extreme seasonal variations in solar irradiance and wind speed particularly relevant to remote Egyptian regions are not fully represented in the simulation scenarios.

Finally, battery degradation is modeled using simplified aging assumptions, which may underestimate long-term capacity fade and replacement costs.

### Future research directions

Building on the identified limitations, future research will focus on the following directions:

1. Uncertainty modeling: The current deterministic framework will be extended by incorporating stochastic or robust optimization techniques to explicitly handle renewable energy intermittency and load demand uncertainty. This will improve the reliability of the sizing and scheduling decisions under real-world variability.
2. Multi-objective optimization: Future work will simultaneously optimize NPC, CO₂ emissions, and battery degradation to identify Pareto-optimal system configurations. This will enable decision-makers to balance economic, environmental, and operational objectives rather than focusing solely on cost minimization.
3. Real-time implementation: The proposed energy management strategy will be validated in a Hardware-in-the-Loop (HIL) environment to assess its real-time performance, communication latency tolerance, and robustness under dynamic operating conditions.
4. Advanced EV integration control: The large-scale integration of electric vehicles may affect power quality and grid stability due to fast charging and frequent connection and disconnection. Future research will investigate advanced control strategies to accommodate increasing EV penetration while maintaining grid stability and power quality.
5. Algorithm benchmarking: A comparative evaluation of the proposed CDO algorithm against other state-of-the-art metaheuristic optimizers will be conducted to assess convergence behavior, solution accuracy, computational efficiency, and robustness across different problem scales.

From a practical perspective, the proposed framework provides a viable solution for remote and off-grid microgrid applications, where reliable and cost-effective energy management is essential. The coordinated integration of BESS, HESS, and EVs enables improved energy utilization, reduced reliance on conventional backup systems, and enhanced operational flexibility. The 44.8% reduction in BESS capacity achieved through multi-storage coordination relative to the HESS-only configuration (Scenario 2) directly translates to lower capital investment and reduced lifecycle costs, reinforcing the economic case for V2G-enabled hybrid microgrids in developing regions.

These findings highlight the potential of multi-storage and V2G integration to support sustainable energy systems, particularly in regions with high renewable energy potential and limited grid infrastructure. As Egypt continues to expand its renewable energy capacity toward its 2035 targets, the proposed hybrid microgrid framework offers a scalable and replicable model for rural electrification and energy security.

**Table 7:** Technical Characteristics of Microgrid System Components^59–65^.

Component	Parameter	Value
PV system	Temperature coefficient	−0.0037
	Efficiency	15%
	Reference temperature	25 °C
	Rated power	1 kW
	Length	1625 mm
	Width	1019 mm
	Thickness	46 mm
	capital cost	7000 $
	O&M cost	20 $/unit-year
	replacement cost	13,885 $
	Lifetime	20 Year
Wind Turbine	Model	Fuhrländer FL 30
	Rated power	30 kW
	Swept area	13 m2
	Cut-in speed	2.5 m/s
	Cut-off speed	25 m/s
	Rated speed	12 m/s
	capital cost	58564.79 $
	O&M cost	3%
	Replacement cost	34553.226 $
	Lifetime	20 Year
Battery Energy Storage	Type	Li_Ion
	Maximum SOC	80%
	Minimum SOC	20%
	Rated capacity per unit	1 kWh
	Battery charging efficiency	90%
	Nominal voltage	600 V
	capital cost	500 $/unit
	Replacement cost	350 $/unit
	O&M cost	10 $/unit
	Lifetime	5 Year
Electrolyzer Unit	Rated Power	1 kW
	Efficiency	75%
	Capital cost	2000 $
	Replacement cost	1500 $
	O&M cost	25 $/unit-year
	Lifetime	20 Year
Hydrogen Tank Unit	Rated Power	1 kW
	Efficiency	95%
	Capital cost	1300 $
	Replacement cost	1200 $
	O&M cost	15 $/unit-year
	Lifetime	20 Year
FC System	Rated Power	1 kW
	Efficiency	50%
	Capital cost	3000 $
	Replacement cost	2500 $
	O&M cost	175 $/unit-year
	Lifetime	5 Year
Inverter Unit	Rated Power	1 kW
	Efficiency	95%
	Capital cost	800 $
	Replacement cost	750 $/kW
	O&M cost	8 $/unit-year
	Lifetime	15 Year
Electric Vehicle	Rated capacity per unit	60 kWh
	capital cost	110 $/kWh
	O&M cost	0 $/kWh
	Replacement cost	110 $/kWh
	Lifetime	10 Year
General Economic Parameters	Interest rate	0.05
	Project lifetime	20 years

Table 7 summarizes the technical and economic parameters used in the proposed model. These parameters represent the operational characteristics and cost assumptions of the system components, including renewable generation units, energy storage systems, and conversion devices.

The selected values are based on commonly reported data in the literature and are chosen to reflect realistic operating conditions. Key parameters such as efficiency, capacity limits, and cost coefficients play a critical role in determining system performance, influencing both energy balance and overall economic outcomes.

These assumptions ensure a consistent and realistic evaluation of the different system configurations considered in this study.

## Data Availability

Data and materials used during the study will be made available upon reasonable request to the corresponding author.
